# Compositional flexibility in irreducible antifluorite electrolytes for next-generation battery anodes†

**DOI:** 10.1039/d4ta07521h

**Published:** 2024-12-23

**Authors:** Victor Landgraf, Mengfu Tu, Zhu Cheng, Alexandros Vasileiadis, Marnix Wagemaker, Theodosios Famprikis

**Affiliations:** a Faculty of Applied Sciences, Delft University of Technology 2629 JB Delft The Netherlands t.famprikis@tudelft.nl m.wagemaker@tudelft.nl

## Abstract

Solid-state batteries currently receive ample attention due to their potential to outperform lithium-ion batteries in terms of energy density when featuring next-generation anodes such as lithium metal or silicon. One key remaining challenge is identifying solid electrolytes that combine high ionic conductivity with stability in contact with the highly reducing potentials of next-generation anodes. Fully reduced electrolytes, based on irreducible anions, offer a promising solution by avoiding electrolyte decomposition altogether. In this study, we demonstrate the compositional flexibility of the disordered antifluorite framework accessible by mechanochemical synthesis and leverage it to discover irreducible electrolytes with high ionic conductivities. We show that the recently investigated Li_9_N_2_Cl_3_ and Li_5_NCl_2_ phases are part of the same solid solution of Li-deficient antifluorite phases existing on the LiCl–Li_3_N tie line with a general chemical formula of Li_1+2*x*_Cl_1−*x*_N_*x*_ (0.33 < *x* < 0.5). Using density functional theory calculations, we identify the origin of the 5-order-of-magnitude conductivity increase of the Li_1+2*x*_Cl_1−*x*_N_*x*_ phases compared to the structurally related rock-salt LiCl phase. Finally, we demonstrate that S_Cl_- and Br_Cl_-substituted analogues of the Li_1+2*x*_Cl_1−*x*_N_*x*_ phases may be synthesized, enabling significant conductivity improvements by a factor of 10, reaching 0.2 mS cm^−1^ for Li_2.31_S_0.41_Br_0.14_N_0.45_. This investigation demonstrates for the first time that irreducible antifluorite-like phases are compositionally highly modifiable; this finding lays the ground for discovery of new compositions of irreducible antifluorite-like phases with even further increased conductivities, which could help eliminate solid-electrolyte decomposition and decomposition-induced Li losses on the anode side in high-performance next-generation batteries.

## Abstract 2

Antifluorite-like solid solutions in the LiCl–LiBr–Li_2_S–Li_3_N phase space combine high ionic conductivity with stability against reduction *versus* lithium metal, opening a promising avenue towards solid-state batteries with next-generation anodes.

## Graphical abstract

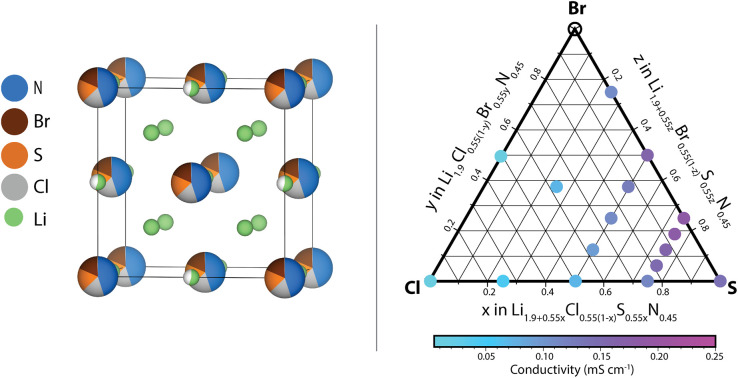

## Introduction

Solid-state batteries have the potential to supersede conventional Li-ion batteries in terms of energy density and safety.^1,2^ Three main advantages of solid electrolytes are as follows. (1) The solid nature of solid electrolytes potentially enables bipolar stacking of individual cells, increasing the overall pack energy density. (2) Solid electrolytes are typically less flammable than liquid equivalents and thus safer. (3) Solid electrolytes are potentially better compatible with high-energy anodes such as silicon or metallic lithium.^1,2^ An initial challenge has been to develop solid electrolytes with sufficiently high ionic conductivities to compete with liquid electrolytes. In recent years, multiple derivatives of the argyrodite and Li_10_GeP_2_S_12_ solid electrolytes have been developed with Li-ion conductivities beyond 10 mS cm^−1^, thus exceeding the conductivities of their liquid equivalents.^3–6^ Nevertheless, the main issue with all known highly conducting (>1 mS cm^−1^) solid electrolytes is that they are generally not (electro-)chemically stable at the high potentials of typical Li-ion battery cathodes nor at low potentials of desirable high-capacity anodes such as silicon or lithium metal. The electrochemical instability of solid electrolytes with electrodes inevitably leads to electrolyte decomposition at the electrolyte–electrode interfaces.^1,7^ Electrolyte decomposition at the electrode interfaces causes lithium loss, formation of resistive interphases and contact loss between solid-electrolyte- and electrode particles, which are all directly linked to battery degradation and failure.^8,9^

This study aims to design solid electrolytes that are highly conducting and thermodynamically stable against the low potentials of desirable high-capacity anodes such as lithium metal and silicon. Such electrolytes would eliminate (electro)chemical degradation on the anode side and thus eliminate complications associated with electrolyte degradation. With regards to thermodynamic stability at low potentials, fully reduced phases become immediately pertinent; *i.e.* phases in which the only cation present is Li and in which all anions are in their lowest permitted formal oxidation state and thus irreducible.^10,11^ Commonly known examples of such irreducible phases are the lithium binaries LiCl, LiBr, Li_2_S, LiI, LiF, Li_3_N and Li_3_P. While these phases all feature thermodynamic stability at the low potentials (<0 V *vs.* Li^+^/Li), they are fraught with low ionic conductivities (<10^−6^ mS cm^−1^), except for Li_3_N, which is reported to have a conductivity of 0.5 mS cm^−1^.^10,12^ New irreducible phases were recently discovered by exploring the tie lines between the above-listed binaries using mechanochemical synthesis. Examples include the Li_2+*x*_S_1−*x*_P_*x*_ phases reported by Szczuka *et al.*^11^ and the Li_2+*x*_S_1−*x*_N_*x*_ phases reported by Landgraf *et al.*;^13^ both systems reaching high conductivities of 0.2 mS cm^−1^. Additionally, the Li_5_NCl_2_ (ref. 10) and Li_9_N_2_Cl_3_ (ref. 14) phases existing on the Li_3_N–LiCl tie line were recently investigated. Li *et al.* demonstrated excellent stability against Li-metal, stability in dry air and high critical-current density for dendrite formation of 10 mA cm^−2^ for the Li_9_N_2_Cl_3_ phase.^14^ Additionally, excellent performance of Li_9_N_2_Cl_3_ in full cells was demonstrated where Li_9_N_2_Cl_3_ is used as an anolyte to protect the Li_2.73_Ho_1.09_Cl_6_ halide electrolyte against a Li metal anode.^14^ These results are promising, however an essential drawback remains the low room-temperature conductivity of Li_9_N_2_Cl_3_ which is reported to be 0.04 mS cm^−1^.^14^ Moreover, the mechanistic origin of the increased conductivity of Li_9_N_2_Cl_3_ phases compared to the structurally related rocksalt LiCl phase has not been established.

The present study develops compositional design strategies to improve the conductivity of Li_9_N_2_Cl_3_ through the following advances:

(1) Synthetically, we demonstrate that the antifluorite framework is compositionally flexible; Li-deficient and Li-excess antifluorite phases can be mechanochemically stabilized. We find that the previously reported Li_5_NCl_2_ and Li_9_N_2_Cl_3_ phases (ref. 15 and 16) are both members of the same solid solution of Li-deficient antifluorite phases on the Li_3_N–LiCl tie line with the general chemical formula of Li_1+2*x*_Cl_1−*x*_N_*x*_ with 0.33 < *x* < 0.5. Additionally, we show that Li_1+2*x*_Cl_1−*x*_N_*x*_ phases are compositionally highly modifiable; we synthesize S_Cl_- and Br_Cl_-substituted analogues boosting the ionic conductivities of Li_1+2*x*_Cl_1−*x*_N_*x*_ phases by an order of magnitude enabling conductivity enhancements up to 0.2 mS cm^−1^ for Li_2.31_S_0.41_Br_0.14_N_0.45_.

(2) Computationally, we explain how introducing nitrogen into the LiCl anionic-framework brings tetrahedral and octahedral lithium sites closer in energy so that vacant sites become energetically accessible for diffusion. Our analysis of diffusion bottlenecks resolving the effect of the local anion coordination shows that nitrogen widens diffusion bottlenecks further facilitating Li diffusion.

(3) Finally, we find through both experiments and computations that the oxidation limit of the irreducible antifluorite-like phases is compositionally tunable and generally higher compared to Li_3_N which may be a critical advantage to stabilize next-generation anodes.

## Results and discussion

**Fig. 1:**
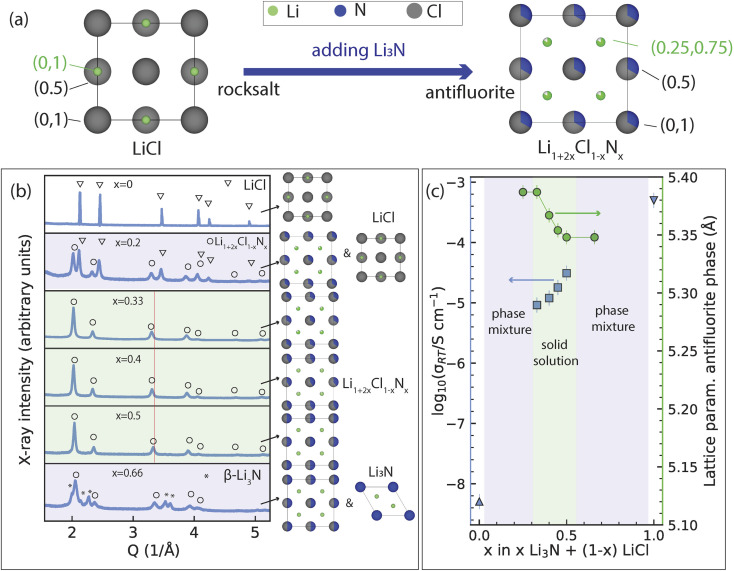
Discovery of a new solid solution on the Li_3_N–LiCl tie line. (a) View along the [001] of the unit cell of rock-salt LiCl and Li-deficient antifluorite Li_1+2*x*_Cl_1−*x*_N_*x*_ phases (exact example of *x* = 0.33 shown). (b) X-ray diffraction pattern of the products of mechanochemically treating *x*Li_3_N + (1 − *x*)LiCl mixtures. In the range (*x* = 0.33 to *x* = 0.5) phase-pure Li-deficient antifluorite structures are obtained. Phases existing at each overall composition are shown next to the diffraction patterns. Red line is guide to the eye. (c) Overall ionic conductivity at 22 °C determined experimentally *via* impedance spectroscopy and the lattice parameter of the antifluorite-like phase for different mixtures of *x*Li_3_N + (1 − *x*)LiCl. Conductivity of (structurally distinct) Li_3_N and LiCl are also shown for reference. Green and purple shading in (b) and (c) indicate solid-solution- and two-phase- regions, respectively.

Our starting point for this work is our previous work on fully reduced electrolytes, showing the conductivity of Li_2_S could be highly improved by dissolving Li_3_N into the antifluorite Li_2_S phase.^13^ Dissolving Li_3_N in Li_2_S results in a series of phases where N and S share sites (Fig. S1†). A solid solution exists with the general chemical formula Li_2+*x*_S_1−*x*_N_*x*_ (0 < *x* < ∼0.5), exhibiting an anion-disordered Li-rich antifluorite crystal structure (Fig. S1†) and showing much higher conductivity (>0.2 mS cm^−1^ for *x* = 0.5) than the Li_2_S host phase (10^−6^ mS cm^−1^).^13^ This motivated our investigation of whether a similar solid solution may be found between Li_3_N and LiCl (schematically illustrated in Fig. 1a), and examine their functional solid–electrolyte properties. To answer this question, Li_3_N and LiCl were mixed in different ratios, followed by a high-energy mechanochemical treatment (ball milling).

### A solid solution of Li-deficient antifluorite phases on the Li_3_N–LiCl tie line

Fig. 1 presents the results of the mechanochemical treatment of *x*Li_3_N + (1 − *x*)LiCl samples and their resulting structure and ionic conductivities. Fig. 1a presents schematically the crystal structures of the parent LiCl rocksalt phase and the target antifluorite phases, confirmed by the powder X-ray diffraction experiments shown in Fig. 1b. Attempting to dissolve small quantities of Li_3_N into LiCl did not result in a pure phase but a mixture of two cubic phases: LiCl and Li_5_NCl_2_. The stoichiometric formula of the latter may alternatively be written as Li_1.66_N_0.33_Cl_0.66_ (*i.e.* Li_1+2*x*_Cl_1−*x*_N_*x*_ with *x* = 0.33), and has been previously reported as a stable phase prepared by conventional solid-state synthesis.^10,15^ Only when the Li_1.66_N_0.33_Cl_0.66_ stoichiometry is reached, having a 2 : 1 LiCl/Li_3_N ratio, a pure Li_1.66_N_0.33_Cl_0.66_ phase is observed (*i.e.* the LiCl phase is not observed in the X-ray diffraction pattern). The mixtures with a higher nitrogen composition than the Li_1.66_N_0.33_Cl_0.66_ phase (Li_1+2*x*_Cl_1−*x*_N_*x*_ with 0.33 < *x* < 0.5) show the same cubic *Fm*3̄*m* diffraction pattern as the *x* = 0.33 phase but with an increasing shift towards larger scattering vector *Q* indicating a decreasing lattice parameter with increasing nitrogen content. When exceeding an overall stoichiometry of Li_2_N_0.5_Cl_0.5_ (*i.e. x* = 0.5) a second phase identified as β-Li_3_N is observed in the diffraction pattern while the lattice parameter of the first phase remains constant. As expected based on the smaller ionic radius of N^3−^ compared to Cl^−^ (1.46 *vs.* 1.81 Å),^17^ the lattice parameter of the Li_1+2*x*_Cl_1−*x*_N_*x*_ phases decreases with increasing nitrogen content, where the linear shift is in accordance with Vegard's law (Fig. 1c). A solid-solution region of cubic Li_1+2*x*_Cl_1−*x*_N_*x*_ phases is thus observed on the on the *x*LiCl–(1 − *x*)Li_3_N tie line for compositions between 0.33 < *x* < 0.5, *i.e.* between the end members Li_1.66_N_0.33_Cl_0.66_ and Li_2_N_0.5_Cl_0.5_.

Rietveld refinements of the X-ray diffractograms (ESI Fig. S2 and Tables S1–4†) show that the crystal structure of the Li_1+2*x*_Cl_1−*x*_N_*x*_ (*x* < 0.33 < 0.5) phases consist in a face-centered-cubic arrangement of the anions where N^3−^ and Cl^−^ share the same site. At the nitrogen-poor boundary of the solid solution (*i.e.* Li_1.66_N_0.33_Cl_0.66_) the tetrahedral interstitials are partially occupied by Li ions (83%).‡‡A small fraction of the Li ions ≤5% may potentially occupy the octahedral sites as detailed in ESI Note 1. With increasing N content the Li content increases to balance the charge, and the tetrahedral sites become increasingly populated until reaching the N-rich solid-solution boundary (*i.e.* Li_2_N_0.5_Cl_0.5_). Consequently, where the Li_2_N_0.5_Cl_0.5_ phase can be described as a (stoichiometric) antifluorite phase (with fully occupied tetrahedral sites), the solid-solution members with (*x* < 0.5) may be described as Li-deficient antifluorite phases (having only partially occupied tetrahedral sites). Further lithium insertion (*x* > 0.5) seems to destabilize the antifluorite, leading to decomposition into a mixture of Li_2_Cl_0.5_N_0.5_ and Li_3_N (Fig. 1b). This is in contrast to the lithium-rich antifluorites Li_2+*x*_S_1−*x*_N_*x*_ which exhibit partial occupation of their octahedral sites in addition to full occupation of their tetrahedral sites (*vide infra*). The structural shift that occurs when going from rock-salt LiCl to Li-deficient antifluorite Li_1.66_N_0.33_Cl_0.66_ entails a significant increase of the cubic lattice parameter from 5.17 Å to 5.39 Å (Fig. 1b), which is likely related to the rearrangement of lithium from the octahedral sites (in the former) to tetrahedral sites (in the latter).

Fig. 1c also shows the ambient-temperature ionic conductivity of the mechanochemically prepared samples as quantified by impedance spectroscopy experiments on pelletized samples which could be invariably fitted by a single bulk-diffusion process (ESI Fig. S3†). The rock-salt LiCl phase has a conductivity of the order of 10^−7^ mS cm^−1^ (Fig. S4†). The conductivity of Li-deficient antifluorite Li_1.66_N_0.33_Cl_0.66_ is significantly higher reaching 0.01 mS cm^−1^, and introducing more nitrogen into the solid solution further increases the conductivity up to 0.03 mS cm^−1^ for Li_2_N_0.5_Cl_0.5_ as shown in Fig. 1c.

We have thus discovered a new solid solution on the LiCl–Li_3_N tie line with the general formula Li_1+2*x*_Cl_1−*x*_N_*x*_ (*x* < 0.33 < 0.5) accessible by mechanochemistry (in contrast to conventional solid state synthesis previously explored in ref. 10 and 15). These phases crystallize in an anion disordered Li-deficient antifluorite-like crystal structure and their conductivity is orders of magnitude higher than the conductivity of rock-salt LiCl. In the next section, we will analyze the mechanistic origin of this 5-order-of-magnitude boost in ionic conductivity.

### Rock-salt LiCl *vs.* Li-deficient antifluorite Li_1+2*x*_Cl_1−*x*_N_*x*_ – origin of the improved conductivity

Fast Li diffusion relies on two prerequisites: (1) Li sites connected by low Li-hop activation energies into a percolating network and (2) a sufficient fraction of vacancies among these Li sites. Vacant Li-sites may be introduced by defects (*e.g.* Frenkel defect pairs) and/or may be synthetically introduced *via* compositional tuning. To investigate Li diffusion in (defect-free) rock-salt LiCl and Li-deficient antifluorite Li_1+2*x*_Cl_1−*x*_N_*x*_ phases we performed *ab initio* molecular dynamics (AIMD) simulations of 2 × 2 × 2 supercells. As done in previous studies, we dissected our AIMD simulations into individual jump events.^10,18–21^ From the frequency of jumps between two sites A and B (*v*_A→B_), we calculate jump-activation energies (jump-*E*_a_) by using eqn (1) and assuming a prefactor frequency (*ν*_0_) of 10^13^ Hz: §§Assuming *v*_0_ = 10^13^ s^−1^ is commonly adopted in the solid electrolyte field.^19,43,44^ Additionally we justify this choice by calculating the average vibration frequency around the equilibrium Li-sites in our AIMD simulations and find it to be 1.0 ± 0.2 10^13^ Hz for 5 different Li_1+2*x*_Cl_1−*x*_N_*x*_ supercells (Table S5).1
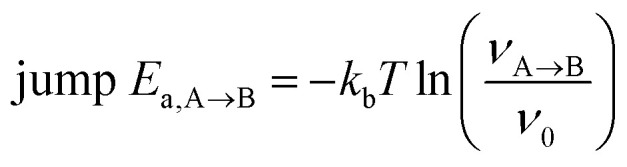
where *k*_b_ is the Boltzmann constant, *T* the temperature in K, *ν*_A→B_ the observed frequency of jumps between sites A and B and jump-*E*_a,A→B_ the jump-activation energy of a jump event from site A to site B. Details on this methodology may be found in ref. 18 and 22. We use these jump-activation energies as a proxy for the local energy barriers. From the difference between the activation energies of the forward and backward jump, the energy difference between two crystallographic positions may be approximated as follows (and illustrated in Fig. S5†):

2 Δ*E*_site_(A, B) = jump-*E*_a,A→B_ − jump-*E*_*a*,B→A_

Δ*E*_site_(tet, oct) may be approximated from eqn (2) as the average of Δ*E*_site_(A, B) for all sites where A and B are tetrahedral and octahedral sites respectively.

**Fig. 2:**
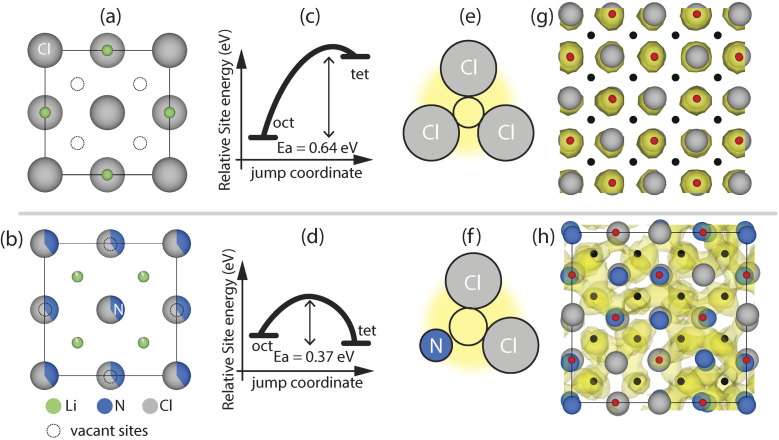
Comparison lithium diffusion in LiCl and Li_1+2*x*_Cl_1−*x*_N_*x*_ phases *via ab initio* molecular dynamics simulations. (a) and (b) View along the [001] of the unit cell of rock-salt LiCl and Li-deficient antifluorite Li_1+2*x*_Cl_1−*x*_N_*x*_ phases. (c) and (d) Schematic illustration of the energy difference and average jump-activation energy between tetrahedral and octahedral sites in rock-salt LiCl and Li-deficient antifluorite Li_1+2*x*_Cl_1−*x*_N_*x*_ phases. (e) and (f) Schematics of the triangular bottlenecks for oct–tet jumps. (g) and (h) LiCl and Li_1.8_Cl_0.6_N_0.4_ supercell with the Li density of a 100 ps AIMD simulation at 1000 K. The Li density cutoff is set to 3% of the maximum value.

Fig. 2 presents the result of our analysis of molecular dynamics of rock-salt LiCl and Li-deficient antifluorite Li_1+2*x*_Cl_1−*x*_N_*x*_ phases.

In LiCl, the octahedral interstitials are preferably occupied over the tetrahedral ones (Fig. 2a), indicating that the former are more stable than the latter. All tetrahedral interstitials in LiCl are vacant thus, in principle, LiCl features a high concentration of vacant interstitials enabling potential diffusion pathways *via* an octahedral–tetrahedral–octahedral jump sequence. |Δ*E*_site_(tet, oct)| is 0.61 ± 0.02 eV in the case of LiCl indicating that tetrahedral sites are on average significantly destabilized by *ca.* 0.6 eV *versus* octahedral sites. The jump-activation energy for the tet–oct jumps in LiCl is low (*ca.* 0.03 eV) suggesting that the tetrahedral site is highly metastable and should arguably more generally be referred to as ‘position’ than ‘site’ as further detailed in ESI Note 2.† For the remaining discussion of this work however this distinction will not be made and the tetrahedral positions in LiCl will also be referred to as sites. One potential reason for the high metastability of tetrahedral Li sites may be the small void space at the tetrahedral site enabling occupation of an ion with a max radius of 0.38 Å (see ESI Note 3†) which is smaller than the Li-ion radius (0.59 Å).^17^ Additionally, oct–tet transitions are sterically hindered in LiCl due to a highly constrained bottleneck with a diameter of 0.8 Å, which requires energetically unfavorably close Li–Cl ion distances and/or lattice distortion to accommodate the passing of Li ions (Fig. 2e). To summarize, in rock-salt LiCl, Li is confined to the octahedral sites and low-activation-energy jumps to vacant sites are not available, rationalizing the absence of diffusion during simulations (localized density in Fig. 2g) and the low RT ion conductivity of LiCl.¶¶Due to the high activation energy required for oct–tet jumps in LiCl and the high metastability of tet sites/positions diffusion in LiCl is likely mediated by Schottky defects just as in NaCl. Accordingly, the charge carrier concentration in LiCl at room temperature is extremely low (∼1 × 10^−9^*c*_Li_ where *c*_Li_ is the Li concentration in LiCl) as the formation energy for Schottky defects is typically ∼1 eV. This low charge carrier concentration additionally contributes to the low ionic conductivity in LiCl.

We now turn our attention to Li-diffusion in the Li_1.66_N_0.33_Cl_0.66_ phase. We performed AIMD simulations on seven different disordered Li_1+2*x*_Cl_1−*x*_N_*x*_ (2 × 2 × 2) supercells (including Li_1.66_N_0.33_Cl_0.66_) to investigate the ion jumps and their jump activation energies present in these phases. Shared site occupations and partial occupancies in Li_1+2*x*_Cl_1−*x*_N_*x*_ phases were treated by random decoration of the Wyckoff 4a (0, 0, 0) position with nitrogen and chlorine and the 8c (0.25, 0.25, 0.25). Li-positions were randomly decorated with Li and vacancies in order to reach the targeted stoichiometry (see Methodology for full computational details). In contrast to LiCl, in the Li-deficient antifluorite Li_1.66_N_0.33_Cl_0.66_ structure the tetrahedral Li sites are occupied, indicating that they are stabilized with respect to the octahedral sites. Li_1.66_N_0.33_Cl_0.66_ features two types of intrinsic vacancies. (1) The tetrahedral sites are partially occupied and (2) the octahedral interstitials are essentially vacant (Fig. 2b).||||Potentially a small fraction of Li ions ≤5% on average may occupy the octahedral sites in which case the octahedral sites as detailed in ESI Note 1. Compared to LiCl the difference in site energy between octahedral and tetrahedral is much smaller, with |Δ*E*_site_(tet–oct)| = 0.07 ± 0.01 eV as compared to |Δ*E*_site_(tet–oct)| = 0.61 ± 0.02 eV in LiCl (Fig. 2c and d). This flat(ter) energy landscape enables low-activation-energy oct–tet jumps and increased bulk diffusion reflected in the much more diffuse Li-density compared to the LiCl case (Fig. 2h) and higher experimentally-measured conductivities of Li_1.66_N_0.33_Cl_0.66_ (Fig. 1c).

**Fig. 3:**
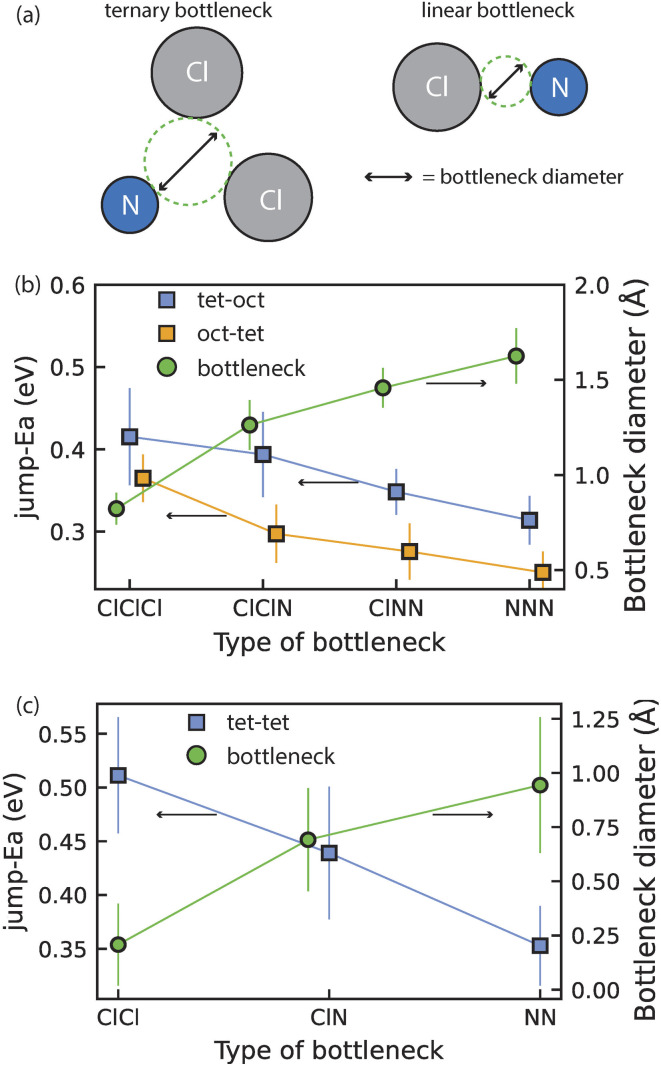
Jump activation energies and bottleneck sizes for ion hops through specific bottlenecks. (a) Schematic of triangular and linear bottlenecks connecting oct–tet and tet–tet site pairs, respectively. (b) Average activation energy of tet–oct and oct–tet jumps for different bottleneck compositions along with the bottleneck size. Error bars are the standard deviation of the distribution of observed bottleneck sizes and activation energies. (c) Same as (b) for tet–tet jumps.

Fig. 3 presents our analysis of ion hopping in disordered Li_1+2*x*_Cl_1−*x*_N_*x*_ as a function of local environment and jump geometry.

From our AIMD simulations we observe that diffusion occurs *via* Li jumps between oct and tet sites through triangular bottlenecks consisting of three anions as well as between tet sites through linear bottlenecks consisting of two anions (Fig. 3a). Li diffusion through triangular and linear bottlenecks is facilitated by the presence of N in the anionic lattice. Because N^3−^ (1.46 Å) anions are considerably smaller than Cl^−^ (1.81 Å) anions, the bottleneck becomes larger, enabling Li diffusion. To quantify this further, we calculated the bottleneck diameters for 5 Li_1.66_N_0.33_Cl_0.66_ DFT relaxed (2 × 2 × 2) supercells (featuring >500 bottlenecks) to account for local distortions that may not be evident in the average crystallographic unit cell. The bottleneck diameters for different bottlenecks as well as the average jump-*E*_a_ for jumps through the respective bottlenecks are shown in Fig. 3. Comparing the bottleneck diameter to the diameter of Li-ions (1.18 Å) is a good proxy for the constraints on Li diffusion; bottlenecks with significantly smaller diameters than 1.18 Å will constrain Li diffusion more than bottlenecks with diameters >1.18 Å. Fig. 3b for instance shows that triangular bottlenecks consisting of three chlorides are considerably smaller (∼0.8 Å) than bottlenecks containing one or more nitrogen (>1.25 Å). Fig. 3b and c show that jumps through nitrogen-containing bottlenecks for oct–tet and tet–tet jumps have lower activation energies indicating easier diffusion through N containing bottlenecks. Consequently, increasing the nitrogen content in the Li_1+2*x*_Cl_1−*x*_N_*x*_ phase, results in more nitrogen-rich, low-activation-energy bottlenecks, explaining the increase in conductivity upon increasing the nitrogen content in the Li_1+2*x*_Cl_1−*x*_N_*x*_ solid solution shown in Fig. 1c.

In conclusion, it is not possible to continuously dissolve Li_3_N into rock-salt LiCl to form Li-excess rock-salt phases as the excess Li ions would occupy tetrahedral sites which are sterically too constrained to accommodate Li ions. However, once a critical amount of Li_3_N (*x* ≥ 0.33) is mixed with LiCl sufficient Li ions are available to stabilize (Li-deficient) antifluorite phases where Li ions occupy tetrahedral sites. The occupation of tetrahedral sites indicates that the tetrahedral sites are more stable than octahedral sites in Li-deficient antifluorite phases. The vacant octahedral sites and the occupied tetrahedral sites are energetically in close proximity (|Δ*E*_site_(tet–oct)| ∼ 0.07 eV) and oct–tet (and tet–tet) transitions are facilitated by the presence of N^3−^ anions in the anionic lattice as the smaller ionic radius of N^3−^ compared to Cl^−^ increases the bottleneck size (Fig. 2f and 3). Consequently, the vacant octahedral sites which are innate to Li-deficient antifluorite phases are thermodynamically and kinetically accessible so that tet–oct jumps may be achieved with jump-*E*_a_ values of 0.37 ± 0.01 eV on average. In contrast, in LiCl the vacant tetrahedral sites are at much larger energies than the occupied octahedral sites (|Δ*E*_site_(tet–oct)| ∼0.6 eV). Additionally, the oct–tet transitions are sterically hindered by small bottlenecks consisting of 3 Cl^−^ ions so that an oct–tet transition has a jump-*E*_a_ value of 0.64 ± 0.01 eV on average. Consequently, the vacant tetrahedral sites which are innate to rock-salt LiCl phases are thermodynamically and kinetically inaccessible explaining the absence of diffusion in simulations and the experimentally obtained low ionic conductivity.

### Comparison between Li-rich antifluorite Li_2+*x*_S_1−*x*_N_*x*_ and Li-deficient antifluorite Li_1+2*x*_Cl_1−*x*_N_*x*_

**Fig. 4:**
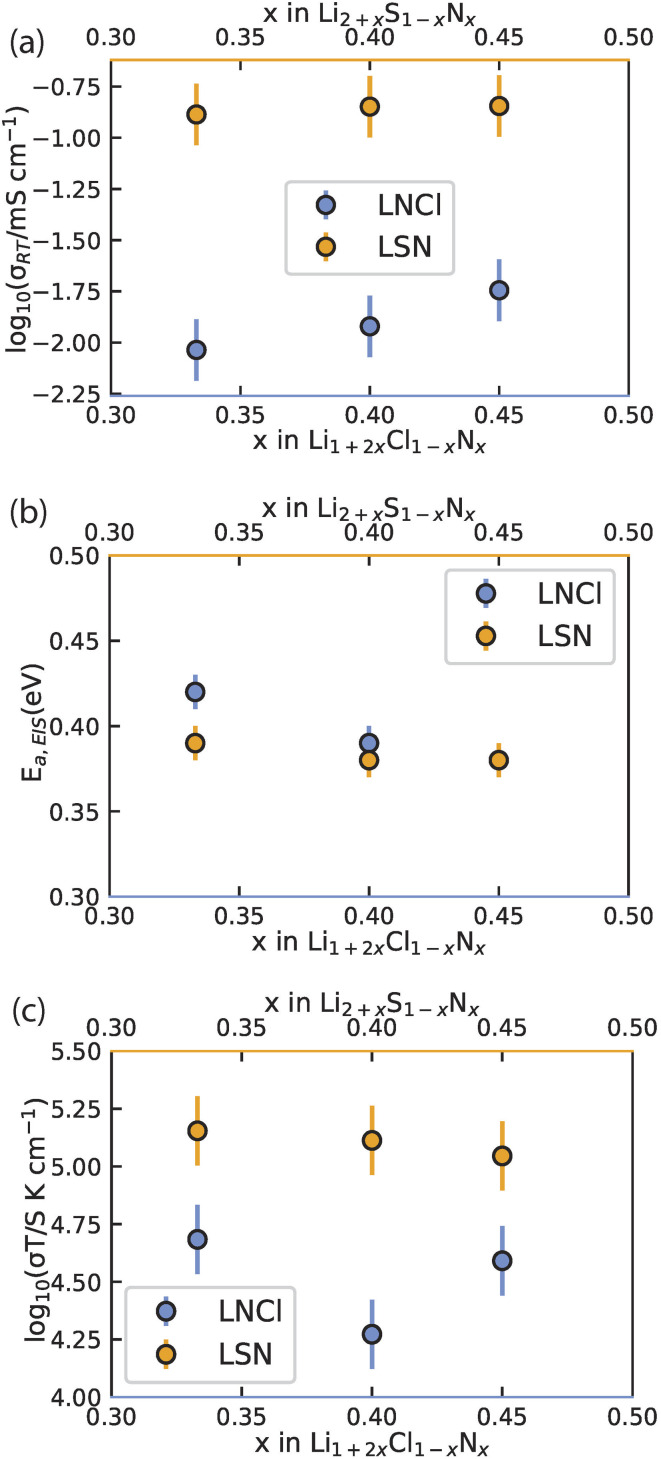
Comparison of the experimentally obtained conductivity, activation energy and Arrhenius prefactor of Li_1+2*x*_Cl_1−*x*_N_*x*_ and Li_2+*x*_S_1−*x*_N_*x*_ phases. (a) Comparison of the conductivity at 22 °C determined experimentally *via* impedance spectroscopy. (b) Comparison of the Arrhenius plot for the activation energy. (c) Comparison of the Arrhenius prefactor. Data for Li_2+_*_x_*S_1−*x*_N_*x*_ originally reported in ref. 13. The Arrhenius fits for (b) are shown in Fig. S6.†

The presently discovered Li_1+2*x*_Cl_1−*x*_N_*x*_ (0.33 < *x* < 0.5) solid-solution phases are a structural analog of the Li_2+*x*_S_1−*x*_N_*x*_ (0 < *x* < ∼0.5) system previously discovered.^13^ Comparing the Li_1+2*x*_Cl_1−*x*_N_*x*_ and Li_2+*x*_S_1−*x*_N_*x*_ phases in the (0.33 < *x* < 0.5) range, where both exist as solid solutions, is insightful in understanding the relationship between structure and Li-ion conductivity. For this we consider three phases of each solid solution, *x* = 0.33, *x* = 0.4 and *x* = 0.45, —representing the lower, center and upper limit of the 0.33 < *x* < 0.5 range, respectively—and compare their ionic conductivity metrics as quantified by variable-temperature impedance spectroscopy. Fig. 4 presents the resulting dependence of the ambient-temperature conductivity, activation energy and conductivity prefactor as a function of composition in Li_1+2*x*_Cl_1−*x*_N_*x*_ and Li_2+*x*_S_1−*x*_N_*x*_.

A notable difference is that the Li_2+*x*_S_1−*x*_N_*x*_ phases are Li-excess antifluorite structures (more than 2 Li per anion), with partial Li occupancy of the octahedral sites (ref. 13) while the Li_1+2*x*_Cl_1−*x*_N_*x*_ phases are Li-deficient antifluorite phases (less than 2 Li per anion) where the tetrahedral sites are partially occupied by Li (Fig. S1 and 2†). Fig. 4a shows that the Li-excess Li_2+*x*_S_1−*x*_N_*x*_ phases have much higher (∼10×) conductivity than the Li-deficient Li_1+2*x*_Cl_1−*x*_N_*x*_ phases, whereas the bulk activation energies, determined by EIS, for the Li_2+*x*_S_1−*x*_N_*x*_ and the Li_1+2*x*_Cl_1−*x*_N_*x*_ phases differ by at most 0.03 eV (Fig. 4b) suggesting that the energy thresholds for diffusion in both systems are similar. However, the difference in the Arrhenius perfactors is large, on average a factor of 7 larger for the phases compared to of the Li_1+2*x*_Cl_1−*x*_N_*x*_ phases (Fig. 4c). This suggests that the origin of the higher conductivity of Li_2+*x*_S_1−*x*_N_*x*_ phases is largely comprised in the Arrhenius prefactor. The larger charge carrier concentration in Li_2+*x*_S_1−*x*_N_*x*_ (and potentially the inducing of concerted motion though we do not explicitly investigate this here) are likely the origin for the larger Arrhenius prefactor of Li-excess Li_2+*x*_S_1−*x*_N_*x*_ antifluorite phases.

### Oxidation limits of Li-rich antifluorite Li_2+*x*_S_1−*x*_N_*x*_ and Li-deficient antifluorite Li_1+2*x*_Cl_1−*x*_N_*x*_ phases

**Fig. 5:**
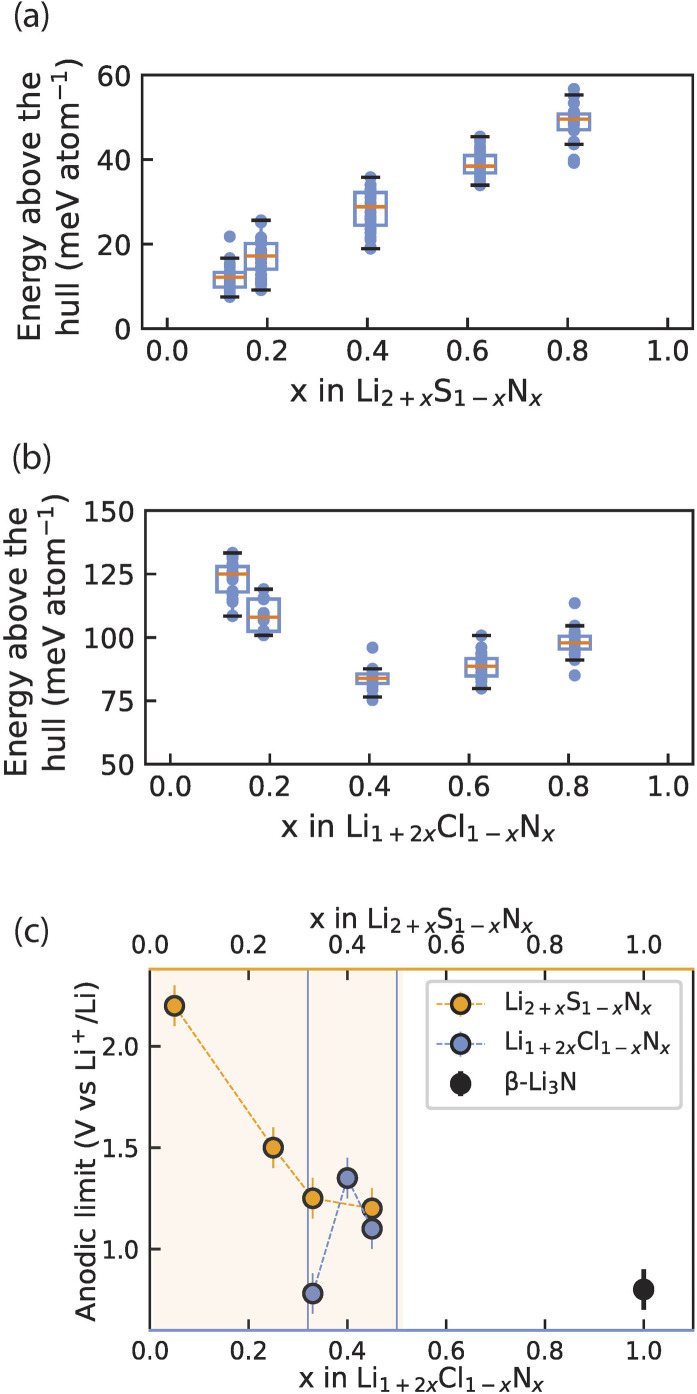
Interplay of phase (meta)stability and anodic limit of irreducible antifluorite phases. (a) and (b) Calculated energy above the hull for 30 Li_2+*x*_N_*x*_S_1−*x*_ and Li_1+2*x*_Cl_1−*x*_N_*x*_ antifluorite supercells at each of several compositions *x*. At each composition above the distribution of energies above the hull is shown as a box plot where the orange line indicates the arithmetic mean and the box indicates the first standard deviation. (c) Experimental anodic limits obtained from LSV for Li_2+*x*_S_1−*x*_N_*x*_, Li_1+2*x*_Cl_1−*x*_N_*x*_ and β-Li_3_N for reference. Orange shading and blue lines denote the solid-solution range for Li_2+*x*_S_1−*x*_N_*x*_ and Li_1+2*x*_Cl_1−*x*_N_*x*_ respectively. Data for Li_2+*x*_S_1−*x*_N*_x_* in (c) originally reported in ref. 13.

We computationally investigated the metastability of Li_2+*x*_S_1−*x*_N_*x*_ and Li_1+2*x*_Cl_1−*x*_N_*x*_ antifluorite-like phases. For compositions *x* = 0.11, 0.17, 0.36, 0.55, 0.72 we built 10 000 random 2 × 2 × 2 antifluorite-like Li_2+*x*_S_1−*x*_N_*x*_ supercells and calculated their electrostatic energies with the formal oxidation states Li^+^, S^2−^ and N^3−^ assigned to the ions. We took the 30 supercells with the lowest electrostatic energies and relaxed the structures using density functional theory. With the energies obtained, the energy above the hull of these phases was calculated using entries of the materials project database (see also ESI Note 4†). Fig. 5 compares the calculated energies with the experimentally obtained stability limits of Li_2+*x*_S_1−*x*_N_*x*_ and Li_1+2*x*_Cl_1−*x*_N_*x*_ as a function of composition.

Fig. 5a shows the average energy above the hull for the Li_2+*x*_S_1−*x*_N_*x*_ phases and shows that increased nitrogen content and Li stuffing increase metastability (*i.e.* higher energy above the hull). Note the energy above the hull was calculated for antifluorite-like Li_2+*x*_S_1−*x*_N_*x*_ phases even at nitrogen contents that cannot be stabilized experimentally (*i.e. x* > 0.55) to clearly demonstrate the effect of Li_3_N dissolution into Li_2_S on phase (meta)stability.

Turning to the Li_1+2*x*_Cl_1−*x*_N_*x*_ phases, the metastability of Li_1+2*x*_Cl_1−*x*_N_*x*_ phases was calculated in an analogous manner to the Li_2+*x*_S_1−*x*_N_*x*_ phases and is shown in Fig. 5b. Note the energy above the hull is calculated for antifluorite-like Li_1+2*x*_Cl_1−*x*_N_*x*_ phases even at nitrogen contents that cannot be stabilized experimentally (*i.e. x* < 0.33 and *x* > 0.5) to clearly demonstrate the effect of nitrogen content on phase (meta)stability. For Li_1+2*x*_Cl_1−*x*_N_*x*_ phases the metastability is high at low and at high nitrogen content. The metastability of Li_1+2*x*_Cl_1−*x*_N_*x*_ phases is lowest around the center of the LiCl–Li_3_N tie line. This non-monotonic trend in the metastability is consistent with the experimentally observed low-N-content and high-N-content boundaries of the Li_1+2*x*_Cl_1−*x*_N_*x*_ (0.33 < *x* < 0.5) solid solution (Fig. 1b and c).

Fig. 5c presents the oxidative stability limits of the Li_2+*x*_S_1−*x*_N_*x*_ and Li_1+2*x*_Cl_1−*x*_N_*x*_ antifluorite-like phases as determined experimentally by linear-sweep voltammetry (ESI Fig. S7 and S8†). The trends in the experimental oxidative limits mirror the trends observed for energy above the hull in Fig. 5a and b: the increased metastability of N-rich antifluorite-like Li_2+*x*_S_1−*x*_N_*x*_ phases is reflected in the oxidation stability which monotonically decreases the higher the nitrogen content and a non-monotonic trend is observed in the oxidation stability of Li_1+2*x*_Cl_1−*x*_N_*x*_ phases with a maximum for *x* = 0.4.

### S_Cl_ and Br_Cl_ substitutions on Li_1+2*x*_Cl_1−*x*_N_*x*_ phases to boost conductivity

**Fig. 6:**
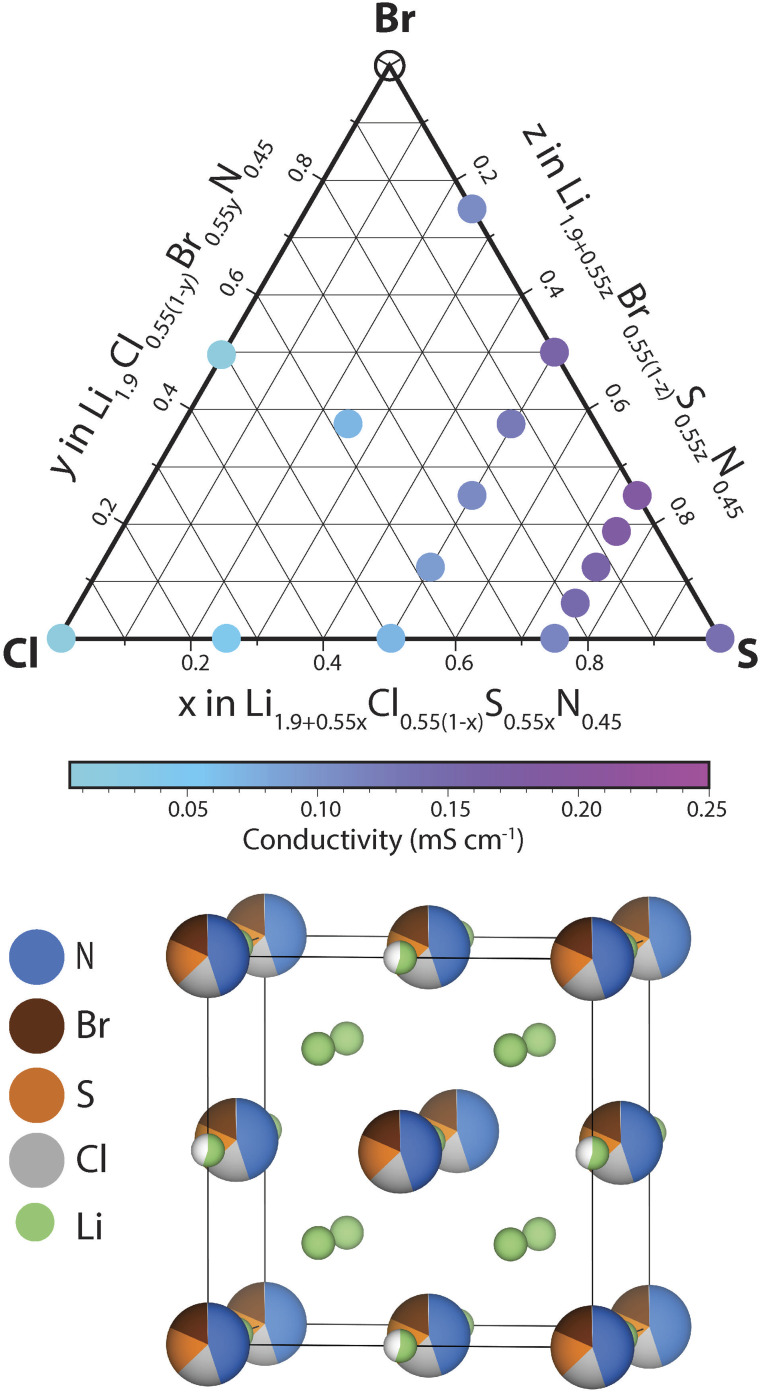
Investigating conductivities in the Li_1.9+0.55*y*_Cl_0.55(1−*y*−*z*)_S_0.55*y*_Br_0.55*z*_N_0.45_ (0 < *y*, *z* < 1) space. Quasi-ternary phase diagram with the Li_1.9_Cl_0.55_N_0.45,_ Li_1.9_Br_0.55_N_0.45_ and the Li_2.45_S_0.55_N_0.45_ compositions at its vertices. The circles represent the phase-compositions that were synthesized as part of this work. Coloured circles signify pure-phase antifluorite-like products. Hollow circles signify no pure-phase antifluorite-like phase product. The colors correspond to experimentally measured ionic conductivities at 22 °C. A schematic unit cell of the phase space investigated is also shown.

In this section we investigate the effect of S_Cl_ and Br_Cl_ substitutions in Li_1+2*x*_Cl_1−*x*_N_*x*_ phases. S_Cl_ substitutions in Li_1+2*x*_Cl_1−*x*_N_*x*_ may increase the number of charge carriers and Br_Cl_ substitutions could increase the lattice parameter facilitating Li diffusion. To narrow down this vast compositional space, the effect of the S_Cl_, Br_S_ and Br_Cl_ substitutions considered at a fixed nitrogen content *x* = 0.45. Thus the phase space we set out to investigate can be expressed by the following solid-solution formula: Li_1.9+0.55*y*_Cl_0.55(1−*y*−*z*)_S_0.55*y*_Br_0.55*z*_N_0.45_ (0 < *y*,*z* < 1). We explored the ionic conductivity of this phase space by synthesizing various compositions in the ternary phase diagram and measuring their ionic conductivity using impedance spectroscopy (provided a single antifluorite-like phase was obtained). The results of this investigation are shown in ESI Fig. S9–S14.†Fig. 6 presents the ionic conductivity of ternary and quaternary Li_1.9+0.55*y*_Cl_0.55(1−*y*−*z*)_S_0.55*y*_Br_0.55*z*_N_0.45_ antifluorite-like samples synthesized as a function of composition in a quasi-ternary phase diagram.

We find that nearly all compositions in the phase space shown in Fig. 6 exist as Li-deficient or Li-excess antifluorite structures. Only highly Br-rich samples where the bromine content exceeds 40% of all anions (that is, values of *z* > 0.75 in Li_1.9+0.55*y*_Cl_0.55(1−*y*−*z*)_S_0.55*y*_Br_0.55*z*_N_0.45_) did not result in phase-pure antifluorite-like phases.

Three main trends may be derived from Fig. 6. (1) S_Cl_ substitutions entail a continuous increase in ionic conductivity. For instance, when substituting Cl in Li_1.9_Cl_0.55_N_0.45_ by S to form Li_2.45_S_0.55_N_0.45_ the conductivity increases by an order of magnitude (from 0.02 to 0.15 mS cm^−1^). This trend can be rationalized based on the increased mobile charge-carrier concentration through charge compensation in replacing S^2−^ by Cl^−^ ions. (2) The second main trend we derive from Fig. 6 is that Br_Cl_ substitutions entail a continuous increase in ionic conductivity. For instance, substituting all Cl in Li_2.175_Cl_0.275_S_0.275_N_0.45_ by Br to form Li_2.175_Br_0.275_S_0.275_N_0.45_ leads to a conductivity increase by a factor of *ca.* 2. This trend may be rationalized by the larger ion radius of Br^−^ compared to Cl^−^: the presence of Br^−^ increases the lattice parameter, which facilitates diffusion through the sterically constrained diffusion bottlenecks. Additionally, the higher polarizability (softness) of Br compared to Cl (ref. 23) may ease diffusion through Br-containing bottlenecks compared to Cl-containing bottlenecks. (3) The third trend we derive from Fig. 6 is that partial Br_S_ substitutions increase ionic conductivities. Unlike the two previous trends, this trend is not monotonic. While partial Br_S_ substitution leads to higher conductivities, carrying the substitution too far leads to decreasing conductivities. This discontinuous trend may be explained as follows. While Br_S_ substitutions increase the lattice parameter, Br_S_ substitutions concurrently reduce the Li concentration to compensate for the lower valence of Br^−^ compared to S^2−^ ions.

In agreement with the trends described above we find that the phase with the highest conductivity is a moderately Br-substituted Li_2.45_S_0.55_N_0.45_ phase – that is a phase with a stoichiometry of Li_2.31_S_0.41_Br_0.14_N_0.45_ reaching a conductivity of *ca.* 0.2 mS cm^−1^ at 22 °C. Overall this investigation demonstrates the high structural and compositional flexibility of the antifluorite framework; Li-deficient and Li-rich antifluorite phases may be stabilized and may feature numerous elements on the anion site.

### Perspectives of irreducible antifluorite-like electrolytes for batteries

Solid-state batteries necessitate high-energy, low-voltage anodes such as Li metal or Li_*x*_Si to supersede conventional Li-ion batteries.^8,24^ However, the best ion conductors known to date—reaching >0.1 mScm^−1^ in conductivity and comprising oxide, sulfide and halide chemistries suffer from electrochemical decomposition <1 V *vs.* Li/Li^+^^7,25–27^—with the exception of garnet oxides which are compatible with Li metal.^28^**Even oxide garnet electrolytes may potentially benefit from protection layers against Li metal.^45,46^ Electrochemical decomposition on the anode side is associated with increased cell resistance, capacity loss, dendrite formation and short-circuiting.^29,30^ A promising strategy to avoid reductive decomposition are bilayer separators comprising a catholyte facing the cathode and an anolyte facing the anode.^1^ Anolytes should be highly-conducting and electrochemically stable at the low potentials of Li metal anodes. Highly conducting fully-reduced phases are thus inherently promising anolyte candidates which has also been demonstrated experimentally.^13,14,31^

High conductivity and reductive stability are not the only criteria suitability criteria of anolytes. The suitability of anolyte layers is system-dependent and multiple factors need to be considered. Mechanical and microstructural properties of solid electrolytes play a key role, for instance in dendrite formation and mechanical degradation in solid-state batteries.^32,33^ Chemical compatibilities of anolytes with the paired catholytes also need to be considered.^10^ Additionally, the oxidation limit of anolytes needs to be considered; for applications *e.g.* with Li_*x*_Si anodes whose operation window ranges from 0.01 V to 1.1 V.^8^ Due to the low oxidation limit of Li_3_N (0.8 V *vs.* Li/Li^+^), Li_3_N would be inert to reduction but not inert to oxidation when in contact with Li_*x*_Si anodes. In contrast, some compositions of the antifluorite-like phases have oxidation limits exceeding 1.1 V (see Fig. 5c) and would be suitable anolytes for Li_*x*_Si anodes as they would be inert to reduction and oxidation. In summary, irreducible electrolytes are promising anolyte candidates. The high compositional and structural flexibility of fully-reduced antifluorite-like phases we demonstrate in this study will enable the further tunability of ionic conductivity, electrochemical stability, mechanical and microstructural properties – essential for functional electrode/electrolyte interfaces in batteries.^32^

## Conclusion

Fully reduced electrolytes based on the antifluorite framework recently received ample attention as their stability against low potentials eliminates performance degradation due to reductive decomposition.^14^ In this study we elucidated the mechanism underlying the increased conductivity in herein discovered irreducible antifluorite-like Li_1+2*x*_Cl_1−*x*_N_*x*_ phases (which includes Li_9_N_2_Cl_3_ ref. 14 and Li_5_NCl_2_ ref. 10 and 15) compared to the structurally similar LiCl phase. Computationally, we find that introducing nitrogen into the LiCl anionic framework brings tetrahedral and octahedral Li sites closer in energy so that vacant sites become energetically accessible for diffusion. Analyzing local diffusion bottlenecks we further showed that nitrogen widens diffusion bottlenecks further facilitating Li diffusion. Experimentally we demonstrated that the antifluorite framework is stoichiometrically flexible; Li-deficient and Li-excess antifluorite phases can be stabilized. We further found that fully reduced antifluorite-like phases have an increased oxidation limit compared to Li_3_N (0.8 V *vs.* Li) which may be critical advantage over Li_3_N (the archetypical fully reduced electrolyte) to stabilize next-generation anodes. Additionally, we showed that Li_1+2*x*_Cl_1−*x*_N_*x*_ phases are compositionally highly modifiable: S_Cl_- and Br_Cl_-substituted analogues were synthesized, boosting the ionic conductivities of Li_1+2*x*_Cl_1−*x*_N_*x*_ phases by an order of magnitude. We demonstrate that ternary and quaternary solid solutions can be synthesized mechanochemically in the LiCl–LiBr–Li_2_S–Li_3_N phase space, unlocking an expansive compositional domain for future materials exploration.

## Methodology

### Synthesis

All preparation steps were performed in an argon atmosphere (H_2_O < 1 ppm, O_2_ < 1 ppm). Li_1+2*x*_Cl_1−*x*_N_*x*_ phases: the synthesis precursors were LiCl (Sigma-Aldrich, 99%) and Li_3_N (Sigma-Aldrich, >99.5%). Stoichiometric amounts of the precursors were milled in a planetary ball mill (Jar : ZrO_2_, 45 mL) with 10 mm ZrO_2_ balls and a ball : powder mass ratio of 30 at 550 rpm for 99 (5 min milling–5 min-pause) cycles. Li_2+*x*_S_1−*x*_N_*x*_ phases (originally reported in ref. 13): the synthesis precursors were Li_2_S (Sigma-Aldrich, 99%) and Li_3_N (Sigma-Aldrich, >99.5%). Stoichiometric amounts of the precursors were milled in a planetary ball mill (Jar : ZrO_2_, 45 mL) with 10 mm ZrO_2_ balls and a ball : powder mass ratio of 30 at 550 rpm for 99 (5 min milling–5 min-pause) cycles.

### Electrochemical characterization

The same procedure was applied to all solid electrolytes (SE) investigated in this work: Li_1+2*x*_Cl_1−*x*_N_*x*_ and Li_2+*x*_S_1−*x*_N_*x*_ and Li_1.9+0.55*y*_Cl_0.55(1−*y*−*z*)_S_0.55*y*_Br_0.55*z*_N_0.45_. Electrochemical Impedance Spectroscopy (EIS): pellets (diameter = 10 mm) of the SE powder samples were pressed (3.2 tons) in custom-made cells. These lab cells consist of an alumina tube and two stainless steel (SS) plungers and an airtight seal. The stainless steel plungers act as current collectors. Solid electrolyte powder is filled in the alumina tube and compressed on both sides with the stainless steel plungers. The cell configuration used was SS|SE|SS. AC impedance was performed with a Metrohm Autolab (AUT86298) in the frequency range 10 MHz to 0.1 Hz with a voltage amplitude of 10 mV. EIS spectra were fitted with a resistor in parallel with a constant phase element (CPE) representing the solid electrolyte and a CPE representing the solid electrolyte–SS interface. RT conductivities were measured in ambient conditions (22 °C in our labs). Linear sweep voltammetry (LSV): LSV measurements were also performed with an Metrohm Autolab (AUT86298). To measure the anodic limit of SE phases, Li|SE|SE–C cells were used. To make the SE–C composite cathode a mixture of SE : Super P with a weight ratio of 0.7 : 0.3 was milled in a planetary ball mill (Jar : ZrO_2_, 45 mL) with 10 mm ZrO_2_ balls and a ball/powder ratio of 30 at 400 rpm for 2 h (5 min milling; 5 min pause). Li|SE|SE-C cells were assembled by pressing a SE pellet (130 mg, 3.2 tons) and subsequently the SE–C composite (15 mg, 3.2 tons) on top. Finally, a Li disk was placed on the opposite side of the SE pellet. The LSV scanning rate was 0.01 mV s^−1^.

### X-ray diffraction

Powder diffraction patterns were collected using Cu Kα X-rays (1.54 Å) on a PANalytical X'Pert Pro X-ray diffractometer. The air sensitive SE probes were loaded into air-tight holders in an Ar-filled glovebox prior to the measurements. GSAS-II^34^ and FullProf^35^ used for LeBail and Rietveld refinements. As a starting point the structure solution of Li_5_NCl_2_ (*i.e.* Li_1.66_Cl_0.66_N_0.33_) was taken. ^15^For Li_1+2*x*_Cl_1−*x*_N_*x*_ phases with higher nitrogen content the nitrogen amount was increased, the chlorine amount reduced and the Li fraction of occupied tetrahedral sites increased according to the synthesized stoichiometry. Based on the initial structure solution of Li_1.66_Cl_0.66_N_0.33_ [ref. 15] all Li ions were assumed in tetrahedral sites though we note that generally a small occupation of the octahedral sites (≤5%) may potentially exist (see ESI Note 1†).

### Computational details

All DFT calculations were performed with the Vienna *ab initio* simulation package VASP with computational settings consistent with those used in the Materials Project database.^36^ Obtaining jump-activation energies for Li_1+2*x*_Cl_1−*x*_N_*x*_ and LiCl. For the generation and analysis of supercells the calculations were done on 7 different 2 × 2 × 2 Li_1+2*x*_Cl_1−*x*_N_*x*_ and one 2 × 2 × 2 LiCl supercells. Because of the shared site occupations and partial occupancies in Li_1+2*x*_Cl_1−*x*_N_*x*_ phases, different atomic arrangements were generated by random decoration of the Wyckoff 4a (0, 0, 0) position with nitrogen and chlorine and the 8c (0.25, 0.25, 0.25) positions were randomly decorated with Li and vacancies. For the generation and analysis of supercells the pymatgen package was used.^37^ For the AIMD simulations the Li pseudopotential was changed from one considering the semicore s electrons as valence (*i.e.* 1s^2^2s^1^ “Li_sv”, which was used for relaxations) to on considering on the 2s electrons (*i.e.* 2s^1^ “Li”) as this enables the use of a lower energy cutoff and vastly improves computational speed. The simulation time was 200 ps for every AIMD simulation. The AIMD simulations were executed at 1000 K. The dissection of AIMD simulations into individual jump events and subsequent analysis of jump frequencies and individual *E*_a,jump_ values was done as first described by de Klerk and Wagemaker^18^ and currently developed as a python package in our group ^39^A comprehensive account can be found in ref. 18 but crucial aspects for the understanding of the reported data is presented here. Calculation of *E*_a_, jump values between two sites: the sites are defined around the 0 K equilibrium positions of the Li ions. At every simulation step it is recorded in which site each Li ion is located or whether it is currently between two sites. From this information the jump frequency between two sites *v*_A→B_ can be calculated according to eqn (3):3
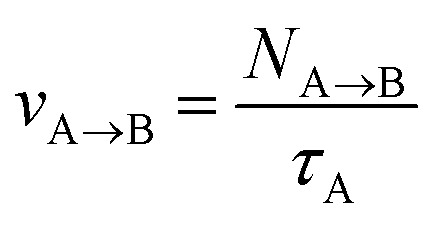
where *v*_A→B_ is the jump frequency for jumps from site A to site B, *N*_A→B_ is the number of recorded jumps from A to B, and *τ*_A_ is the time of occupation of site A. *E*_a,jump_ is then obtained from eqn (1). This analysis can be done with the *gemdat* ref. 38 python package currently developed in our group. To obtain a picture as complete as possible for the jump processes in the disordered Li_1+2*x*_Cl_1−*x*_N_*x*_ phases we executed the AIMD simulations on 5 supercells that together feature all of the possible site-bottleneck-site permutations in Li_1+2*x*_Cl_1−*x*_N_*x*_ phases. A table with jump-*E*_a_ values is provided Table S7.†

Stability calculations for Li_1+2*x*_Cl_1−*x*_N_*x*_ and for Li_2+*x*_S_1−*x*_N_*x*_. 10 000 2 × 2 × 2 supercells were generated by randomly decorating the Wyckoff 4a (0, 0, 0) position with nitrogen and chlorine. The 8c (0.25, 0.25, 0.25) positions were also randomly decorated with Li and vacancies according to the targeted stoichiometry. Then formal charges of −3, −1, and +1 were assigned to nitrogen, chlorine and Li, respectively and the electrostatic energy (‘Ewald energy’) was calculated *via pymatgen*. Subsequently, out of the Li_1+2*x*_Cl_1−*x*_N_*x*_ structures, 30 with the lowest Ewald energy were taken and relaxed by DFT. The energies obtained from DFT were used to calculate the energy of the hull of the Li_1+2*x*_Cl_1−*x*_N_*x*_ phases, corrections from the materials project data base were applied and the energies for the end-member phases (Li_3_N and Li_2_S) were also obtained from the materials project data base (see also ESI Note 4†).^36^

## Data availability

The data that support the findings of this study and the code to reproduce the results shown in the paper are openly available in 4TU. ResearchData at https://doi.org/10.4121/fcb46e92-06cd-4241-a97b-3390d6dc1f70. We used python version 3.10 and the following python packages: numpy^39^, gemdat^38^, matplotlib^40^, pymatgen^37^.

## Author contributions

The study was conceptualized by V. L. Simulation data were acquired by V. L. Experimental data were acquired by V. L., M. T. and Z. C. Data analysis and interpretation were done by V. L., T. F., M. T. and M. W. Writing and editing of the draft were done by V. L., T. F., M. W. The funding for this study was acquired by M. W. and T. F. The project was supervised by T. F. and M. W. All authors have approved the submitted version of the manuscript.

## Conflicts of interest

There are no conflicts to declare.

## Supplementary Material

TA-013-D4TA07521H-s001

TA-013-D4TA07521H-s002

TA-013-D4TA07521H-s003

TA-013-D4TA07521H-s004

TA-013-D4TA07521H-s005
