# Supplementary material for: Compositional flexibility in irreducible antifluorite electrolytes for next-generation battery anodes
Source: J Mater Chem A Mater. 2024 Dec 23;13(5):3562–74. doi: 10.1039/d4ta07521h (PMC11665506; doi:10.1039/d4ta07521h)
Supplement: TA-013-D4TA07521H-s001 [file TA-013-D4TA07521H-s001.pdf]

Supplementary Information (SI) for Journal of Materials Chemistry A.
This journal is © The Royal Society of Chemistry 2024

Compositional flexibility in irreducible antifluorite electrolytes for next-generation battery anodes

Victor Landgraf, Mengfu Tu, Zhu Cheng, Alexandros Vasileiadis, Marnix Wagemaker*, Theodosios Famprikis*

Faculty of Applied Sciences, Delft University of Technology, 2629 JB Delft, The Netherlands

* t.famprikis@tudelft.nl; m.wagemaker@tudelft.nl

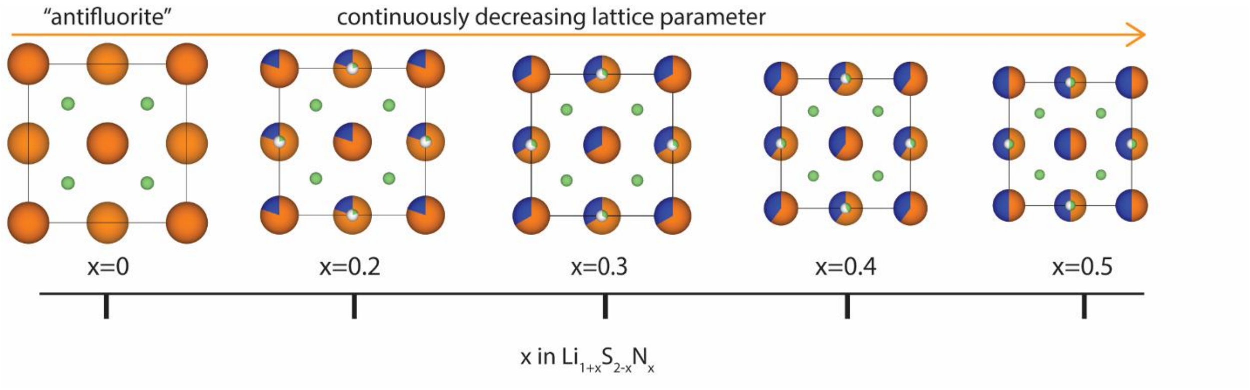

Figure S1. Schematic Illustration of the solid solution existing on the Li_2S-Li_3N tie line, that is the $Li_{2+x}S_{1-x}N_x$ phases reported in ref [1].

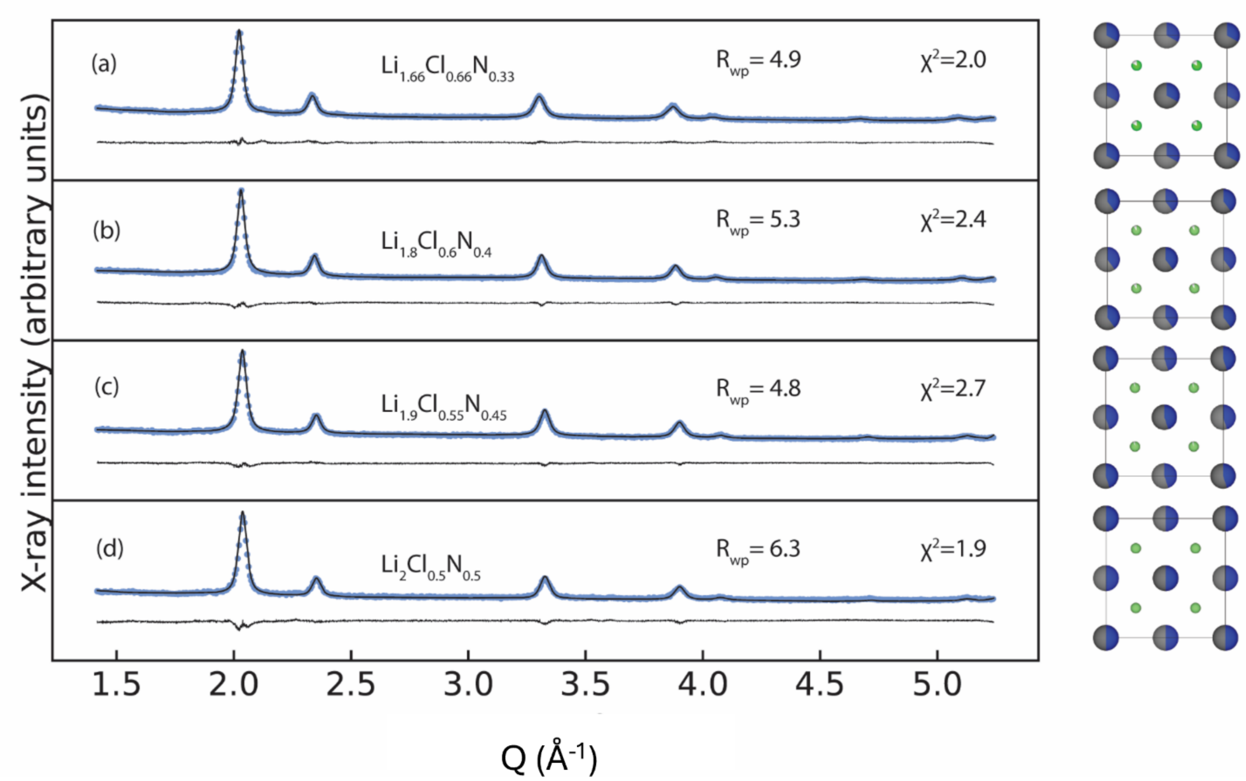

Figure S2.Rietveld refinements of the X-ray diffraction patterns of different $Li_{1+2x}S_{1-x}N_x$ phases along with an illustration of the respective structures solutions on the right. (a) x=0.33. (b) x=0.4, (c) x=0.45, (d) x=0.5.

Table S1. Structure solution of the $Li_{1.66}Cl_{0.66}N_{0.33}$ structure. R_{wp} = 4.9, χ^2 = 2.0, Lattice parameter: a = 5.387(1) Å. The rather large B_{iso} value for Li originate from displacive relaxations off the centre of tetrahedral Li sites with a mix of N and Cl at their corners. [2]

Atom	x	y	z	Wyckoff	Occupancy	B_{iso}
Li	0.25	0.25	0.25	8c	0.833	4.41183(7)
N	0	0	0	4a	0.333	0.99577(8)
Cl	0	0	0	4a	0.666	0.99577(8)

Table S2. Structure solution of the $Li_{1.8}Cl_{0.6}N_{0.4}$ structure. R_{wp} = 5.3, χ^2 = 2.4. Lattice parameter: a = 5.368(1) Å. The rather large B_{iso} value for Li originate from displacive relaxations off the centre of tetrahedral Li sites with a mix of N and Cl at their corners. [2]

Atom	x	y	z	Wyckoff	Occupancy	B_{iso}
Li	0.25	0.25	0.25	8c	0.9	7.72856(7)
N	0	0	0	4a	0.4	0.77151(9)
Cl	0	0	0	4a	0.6	0.77151(9)

Table S3. Structure solution of the $Li_{1.9}Cl_{0.55}N_{0.45}$ structure. R_{wp} = 4.8, χ2 = 2.7 Lattice parameter: a = 5.354(1) Å. The rather large B_{iso} value for Li originate from displacive relaxations off the center of tetrahedral Li sites with a mix of N and Cl at their corners. [2]

Atom	x	y	z	Wyckoff	Occupancy	B_{iso}
Li	0.25	0.25	0.25	8c	0.95	8.45905(7)
N	0	0	0	4a	0.45	0.99576(6)
Cl	0	0	0	4a	0.55	0.99576(6)

Table S4. Structure solution of the $Li_2Cl_{0.5}N_{0.5}$ structure. R_{wp} = 6.5, χ^2 = 1.8 Lattice parameter: a = 5.348(1) Å. The rather large B_{iso} value for Li originate from displacive relaxations off the center of tetrahedral Li sites with a mix of N and Cl at their corners. [2]

Atom	x	y	z	Wyckoff	Occupancy	B_{iso}
Li	0.25	0.25	0.25	8c	1.00	11.69183(6)
N	0	0	0	4a	0.50	1.37784(8)
Cl	0	0	0	4a	0.50	1.37784(8)

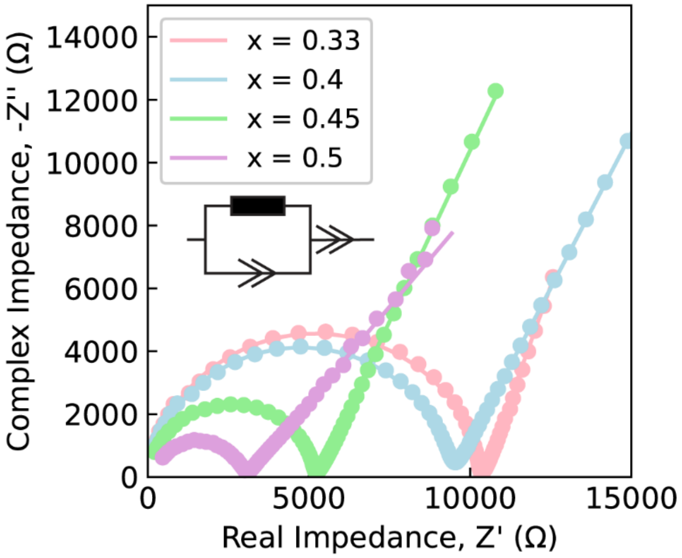

Figure S3. Room temperature electrochemical impedance spectroscopy (EIS) of different $Li_{1+2x}Cl_{1-x}N_x$ phases. The equivalent circuit fitted is shown in the inset of the figure.

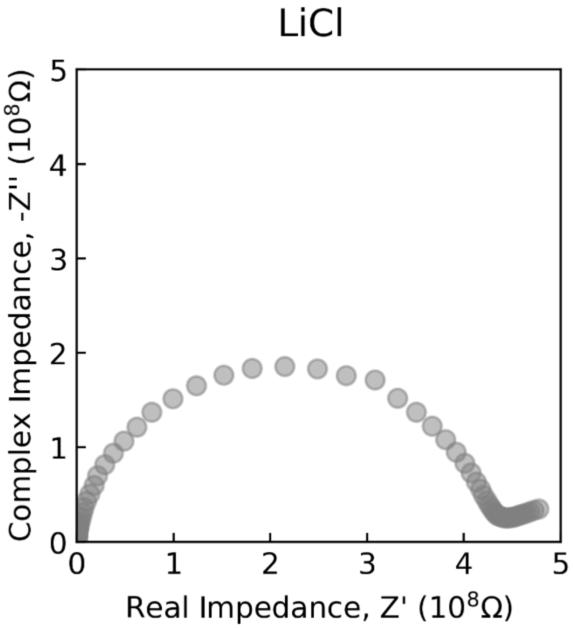

Figure S4. Room temperature electrochemical impedance spectroscopy (EIS) of different LiCl phase corresponding to a conductivity on the order of 10^{-10} S cm^{-1}.

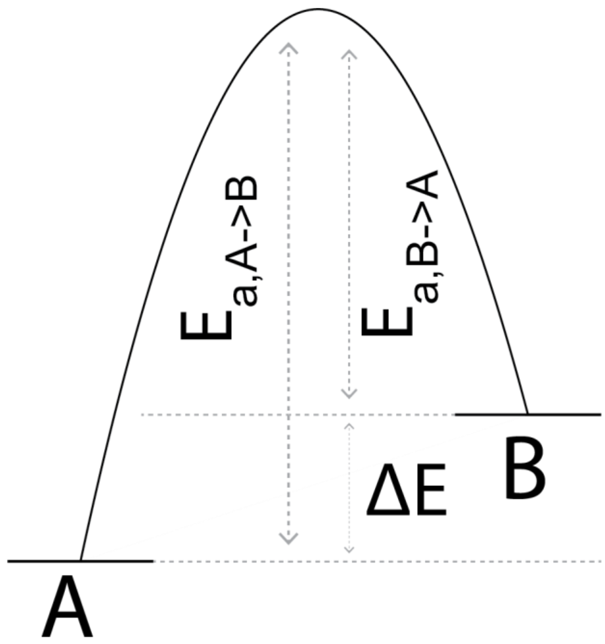

Figure S5. Schematic of the energy profile for a Li ion jump from site A to site B. This figure demonstrates that if Ea, A->B > Ea, B->A then site A is more stable than site B by ΔE.

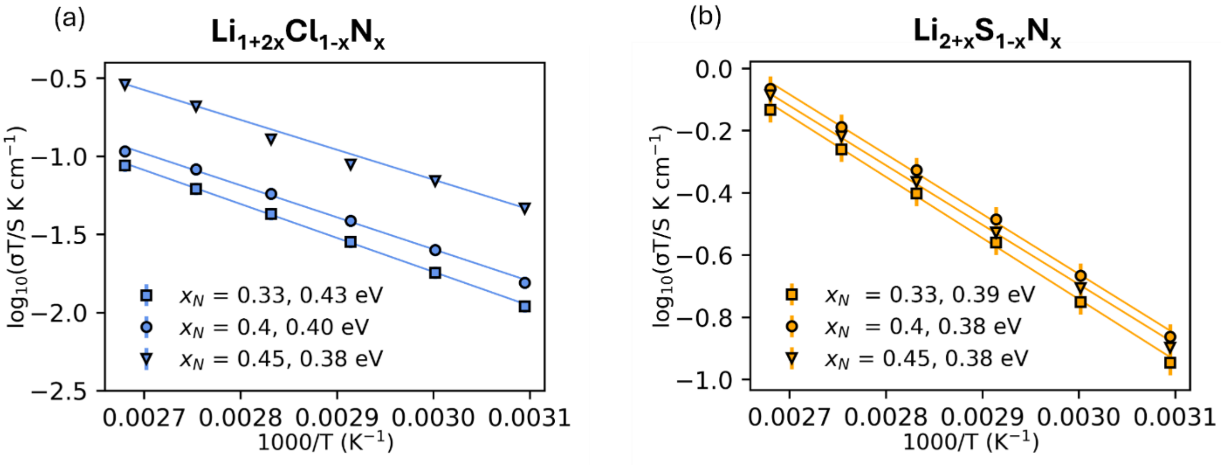

Figure S6. Arrhenius fits for the investigated antifluorite-like irreducible phases. (a) Arrhenius plots for $Li_{1+2x}Cl_{1-x}N_x$ from 50 to 100 C for x=0.33, x0.4, and x=0.45. (b) Arrhenius plot for $Li_{2+x}S_{1-x}N_x$ for x=0.33, x0.4, and x=0.45. Data for $Li_{2+x}S_{1-x}N_x$ in (b) originally reported in ref. [1].

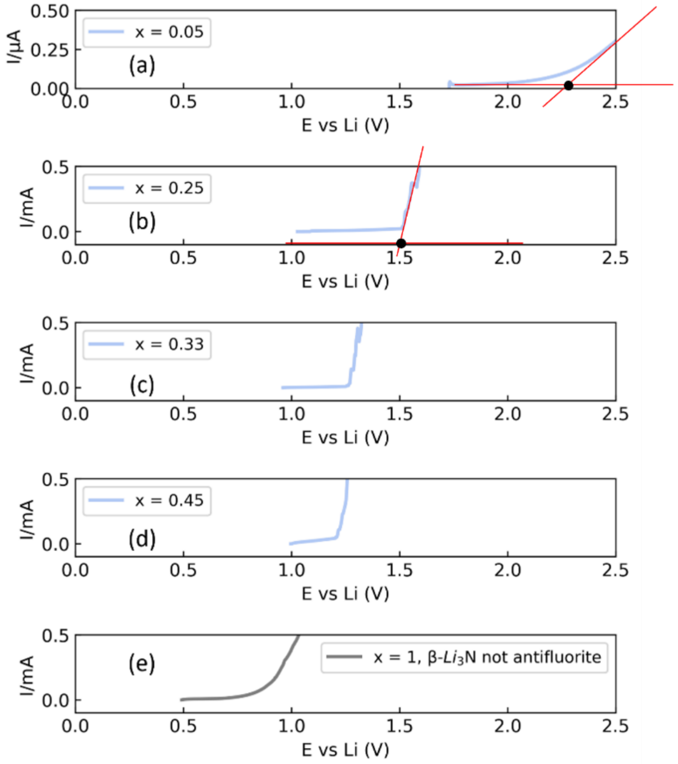

Figure S7. LSV of Li/$Li_{2+x}S_{1-x}N_x$/ $Li_{2+x}S_{1-x}N_x$–C cells and of a Li/Li_3N/ Li_3N-C cell. We show that increasing the nitrogen content progressively decreases the oxidation limit. Scan rate 0.01 mV s^{-1}. The red lines in (a) and (b) show how the oxidation onset is obtained from the LSV measurements. We accord an uncertainty of ±0.1 V to the onsets determined in this way. The red lines are only shown for (a) and (b) but this approach was adopted to all LSV measurements. Data originally reported in ref. [1]

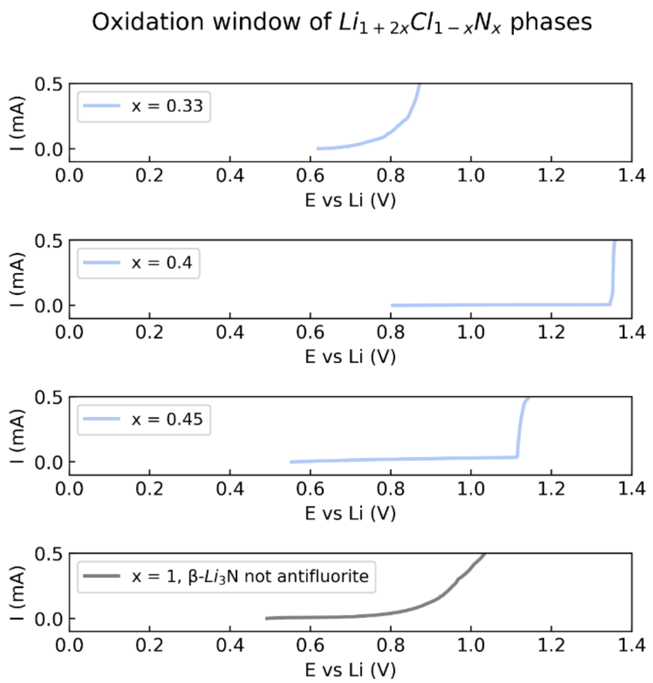

Figure S8. LSV of Li/$Li_{1+2x}Cl_{1-x}N_x$/ $Li_{1+2x}Cl_{1-x}N_x$ –C cells and of a Li/Li_3N/ Li_3N-C cell. We show that there is a discontinuous trend. Increasing the nitrogen content from x=0.33 to x=0.4 increases the oxidation limit. Further increasing the N content decreases the oxidation limit increasing the nitrogen content progressively decreases the oxidation limit. This may be explained by the increasing metastability of the Li-deficient antifluorite $Li_{1+2x}Cl_{1-x}N_x$ phases at both the upper-N and lower-N boundary of the solid solution. Scan rate 0.01 mV s^{-1}. The oxidation onsets were determined following the approach shown in Figure S6 (a) and (b).

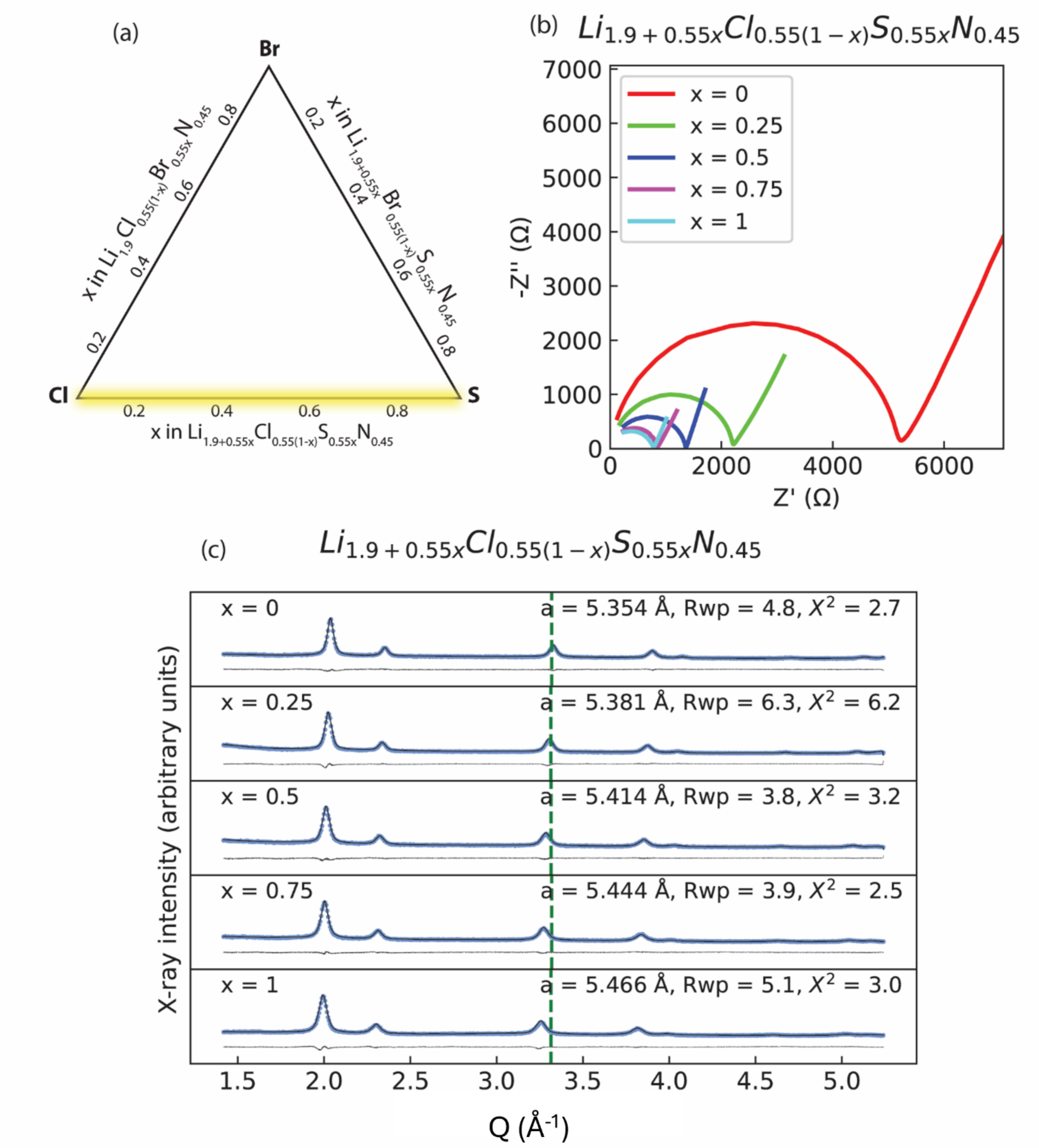

Figure S9. (a) highlighting which phases are shown in "b" and "c". (b) Room temperature electrochemical impedance spectroscopy (EIS) of different $Li_{1.9+0.55x}Cl_{0.55(1-x)}S_{0.55x}N_{0.45}$ phases. (c) X-ray diffraction pattern and Rietveld refinements of the different phases $Li_{1.9+0.55x}Cl_{0.55(1-x)}N_{0.45}$. Dotted green line is guide to the eye to easily see the peak shift originating from different lattice parameters.

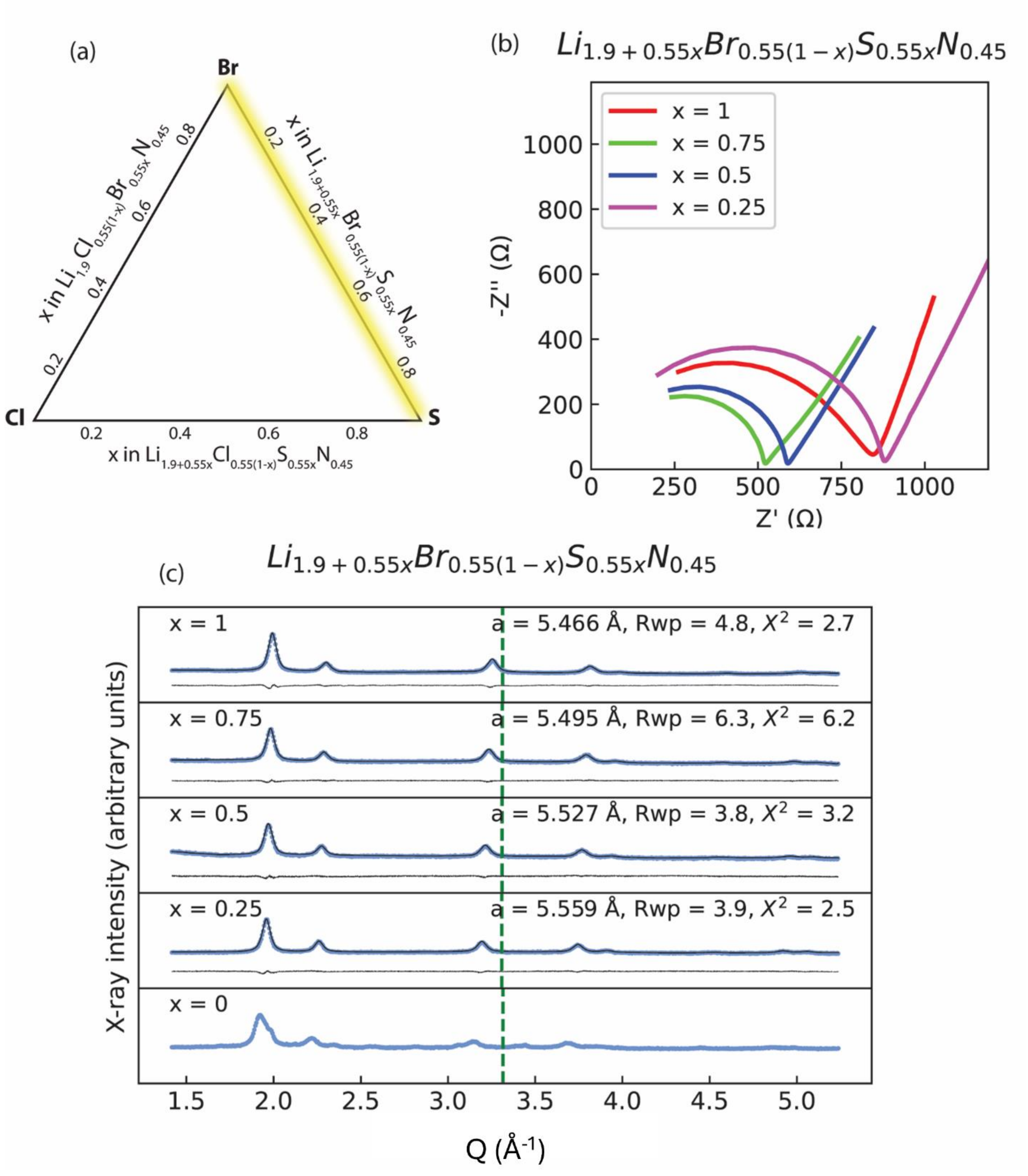

Figure S10. (a) highlighting which phases are shown in "b" and "c". (b) Room temperature electrochemical impedance spectroscopy (EIS) of different $Li_{1.9+0.55x}Br_{0.55(1-x)}S_{0.55x}N_{0.45}$ phases. (c) X-ray diffraction pattern and Rietveld refinements of the different phases $Li_{1.9+0.55x}Br_{0.55(1-x)}N_{0.45}$. At x=0 an impure phase was obtained. The antifluorite structure can thus not be stabilized at this composition. Dotted green line is guide to the eye to easily see the peak shift originating from different lattice parameters.

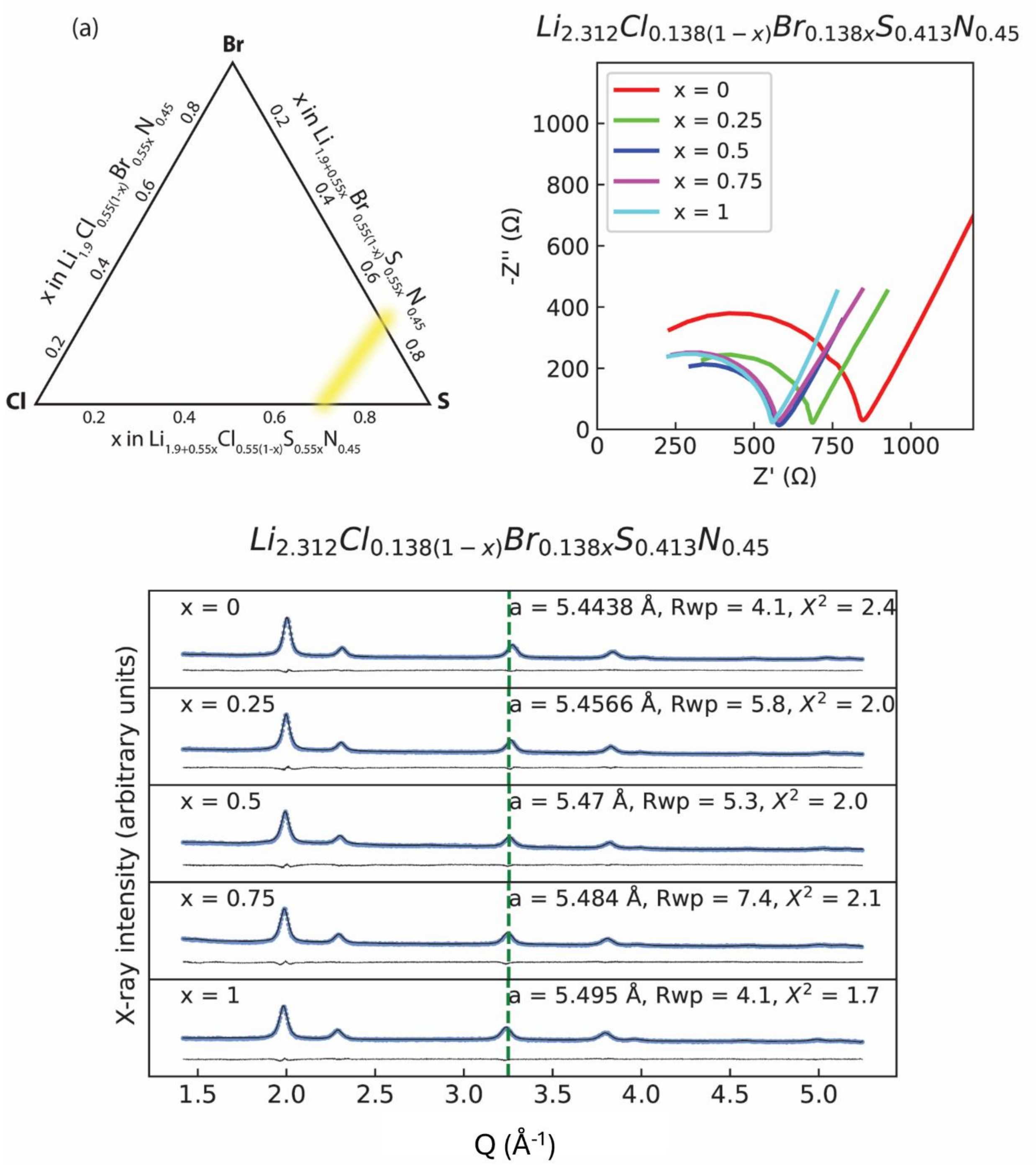

Figure S11. (a) highlighting which phases are shown in "b" and "c". (b) Room temperature electrochemical impedance spectroscopy (EIS) of different $Li_{2.31}Br_{0.138x}Cl_{0.138(1\text{-}x)}S_{0.55x}N_{0.45}$ phases. (c) X-ray diffraction pattern and Rietveld refinements of the different $Li_{2.31}Br_{0.138x}Cl_{0.138(1\text{-}x)}S_{0.55x}N_{0.45}$ phases. Dotted green line is guide to the eye to easily see the peak shift originating from different lattice parameters.

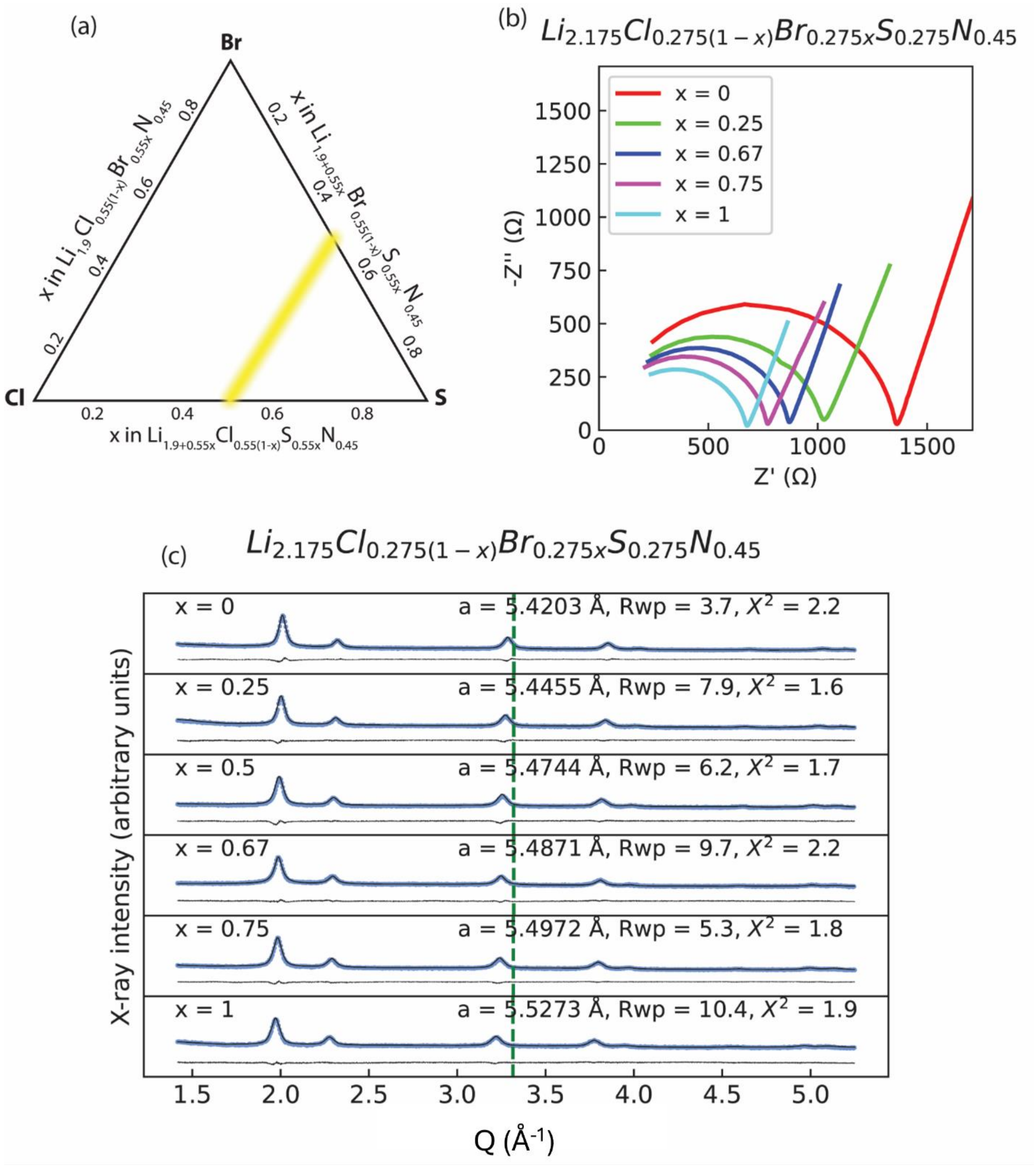

Figure S12. (a) highlighting which phases are shown in "b" and "c". (b) Room temperature electrochemical impedance spectroscopy (EIS) of different $Li_{2.175}Br_{0.275x}Cl_{0.275(1-x)}S_{0.55x}N_{0.45}$ phases. (c) X-ray diffraction pattern and Rietveld refinements of the different $Li_{2.31+0.55x}Br_{0.275x}Cl_{0.275(1-x)}S_{0.55x}N_{0.45}$ phases. Dotted green line is guide to the eye to easily see the peak shift originating from different lattice parameters.

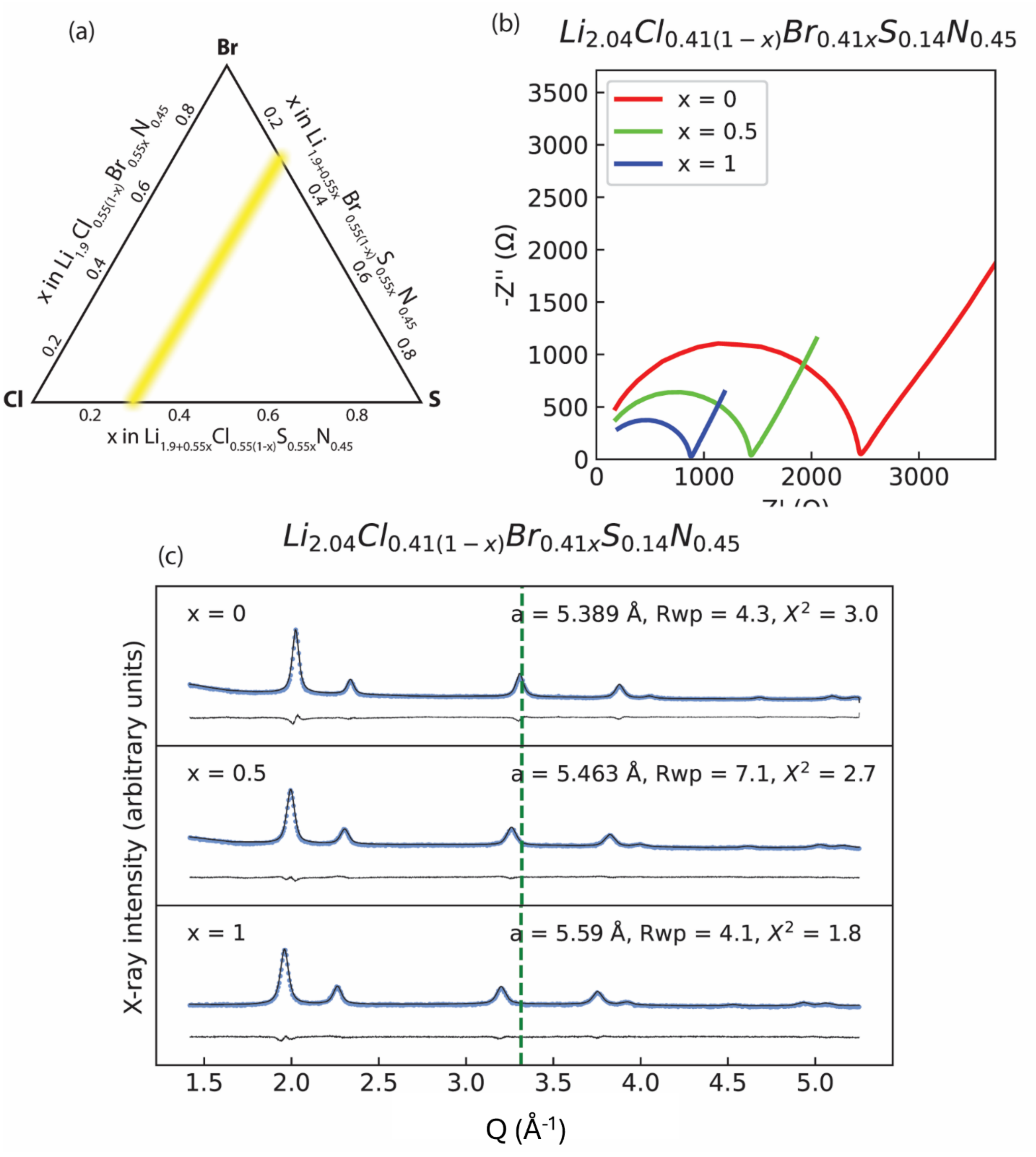

Figure S13. (a) highlighting which phases are shown in "b" and "c". (b) Room temperature electrochemical impedance spectroscopy (EIS) of different $Li_{2.04}Br_{0.41x}Cl_{0.41(1-x)}S_{0.55x}N_{0.45}$ phases. (c) X-ray diffraction pattern and Rietveld refinements of the different $Li_{2.04}Br_{0.41x}Cl_{0.41(1-x)}S_{0.55x}N_{0.45}$ phases. Dotted green line is guide to the eye to easily see the peak shift originating from different lattice parameters.

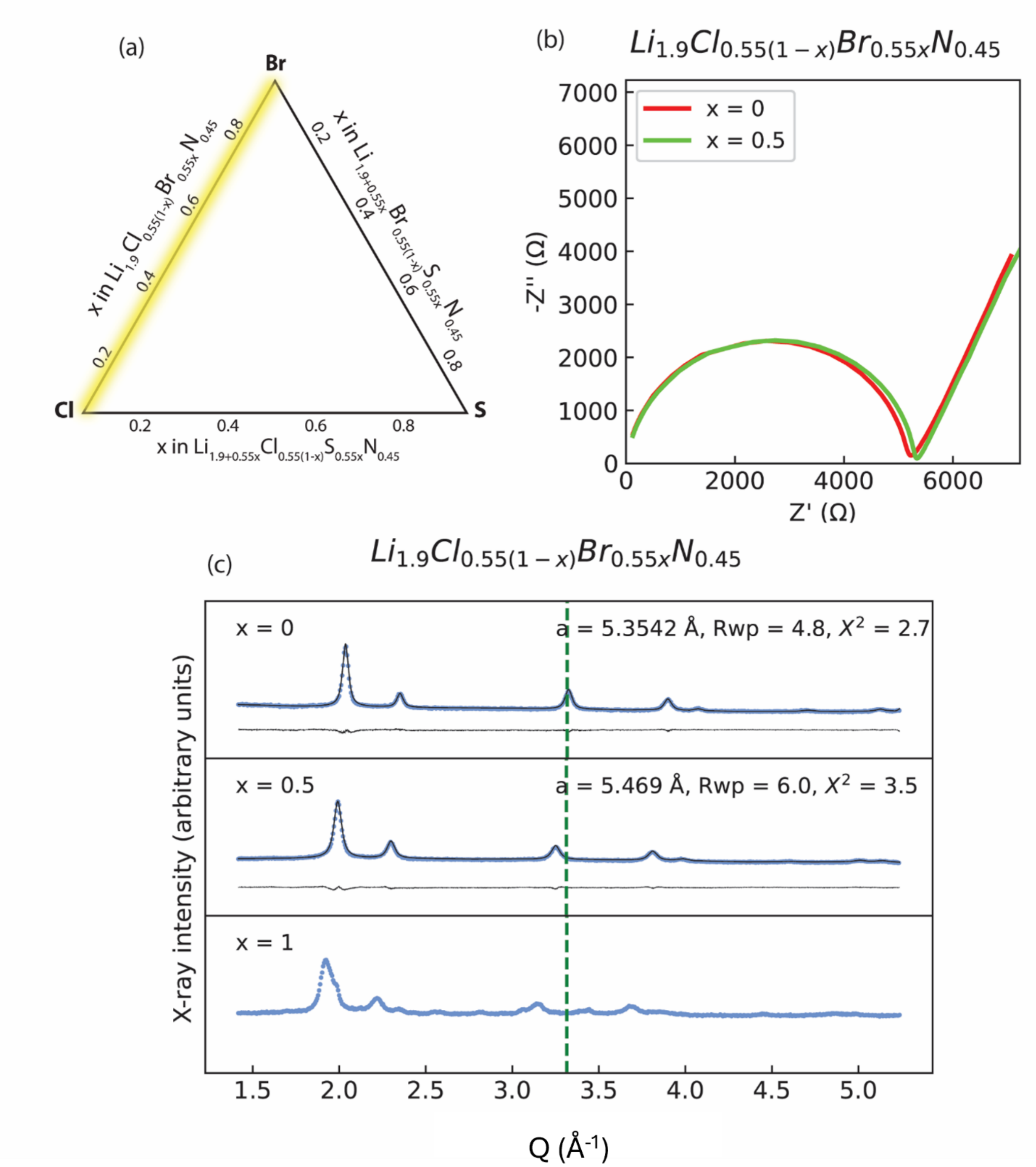

Figure S14. (a) highlighting which phases are shown in “b” and “c”. (b) Room temperature electrochemical impedance spectroscopy (EIS) of different $Li_{1.9}Br_{0.55x}Cl_{00.55(1-x)}S_{0.55x}N_{0.45}$ phases. (c) X-ray diffraction pattern and Rietveld refinements of the different $Li_{2.04}Br_{0.41x}Cl_{0.41(1-x)}S_{0.55x}N_{0.45}$ phases. At x=1 an impure phase was obtained. The antifluorite structure can thus not be stabilized at this composition. Dotted green line is guide to the eye to easily see the peak shift originating from different lattice parameters.

Table S5. Attempt frequency obtained from 5 $Li_{1+2x}N_xCl_{1-x}$ (2x2x2) supercells at 1000 K.

**Supercell $Li_{2+x}N_xCl_{1-x}$ Supercell**	**Attempt frequency (10^{13} Hz)**	**Standard deviation (10^{13} Hz)**
1	1.0	0.21
2	1.0	0.22
3	1.0	0.22
4	1.0	0.23
5	1.0	0.24

Table S6. Lattice parameter (experimental obtained from refinements) of different antifluorite-like phases. The lattice parameters for $Li_{2+x}S_{1-x}N_x$ phases are obtained from previous work (ref 1).

x	Lattice params of antifluorite-like structure (Å)	
	$Li_{1+2x}N_xCl_{1-x}$	$Li_{2+x}N_xS_{1-x}$
0.33	5.387	5.521
0.4	5.367	5.491
0.45	5.354	5.471

Table S7. Table with the average jump-Ea values for each jump type existing in $Li_{1+2x}N_xCl_{1-x}$ phases obtained from AIMD simulations of $Li_{1+2x}N_xCl_{1-x}$ supercells. The total uncertainty on the individual jump-Ea values comprises uncertainty from convergence and uncertainty on the mean value.

Jump type	Average jump-Ea (eV)	Uncertainty on Mean (eV)	Uncertainty from convergence (eV)	Total uncertainty (eV)
N_1Cl_5-Cl_4(ClClCl)	0.282	0.004	0.004	0.008
N_1Cl_5-N_1Cl_3(ClClN)	0.249	0.003	0.001	0.005
N_5Cl_1-N_3Cl_1(NNN)	0.289	0.006	0.001	0.008
N_5Cl_1-N_2Cl_2(ClNN)	0.291	0.006	0.007	0.013
N_1Cl_5-N_1Cl_3(ClClCl)	0.363	0.007	0.014	0.021
N_4Cl_2-N_2Cl_2(ClNN)	0.288	0.004	0	0.004
N_4Cl_2-N_1Cl_3(ClClN)	0.315	0.012	0.014	0.026
N_4Cl_2-N_2Cl_2(ClClN)	0.336	0.007	0.005	0.012
N_6-N_3Cl_1(NNN)	0.243	0.003	0.004	0.007
N_2Cl_4-N_1Cl_3(ClClN)	0.268	0.003	0.001	0.004
N_2Cl_4-N_2Cl_2(ClClN)	0.281	0.004	0.001	0.004
N_3Cl_3-N_2Cl_2(ClClN)	0.327	0.004	0.003	0.007
N_3Cl_3-N_1Cl_3(ClClN)	0.287	0.004	0.006	0.009
N_3Cl_3-N_2Cl_2(ClNN)	0.264	0.003	0.001	0.004
N_3Cl_3-N_3Cl_1(ClNN)	0.246	0.002	0.001	0.003
N_3Cl_3-Cl_4(ClClCl)	0.362	0.008	0.012	0.02
N_1Cl_5-N_2Cl_2(ClClN)	0.289	0.004	0.002	0.006
N_2Cl_4-Cl_4(ClClCl)	0.336	0.005	0.008	0.013
N_4Cl_2-N_3Cl_1(ClNN)	0.289	0.007	0.005	0.013
N_3Cl_3-N_4(NNN)	0.24	0.005	0.001	0.006
N_3Cl_3-N_1Cl_3(ClClCl)	0.457	0	0.457	0.04

N_4Cl_2-N_3Cl_1(NNN)	0.267	0.006	0.003	0.009
N_4Cl_2-N_4(NNN)	0.247	0.011	0.004	0.015
N_2Cl_4-N_2Cl_2(ClNN)	0.233	0.004	0.002	0.006
N_2Cl_4-N_3Cl_1(ClNN)	0.244	0.004	0.003	0.007
N_2Cl_4-N_1Cl_3(ClClCl)	0.358	0.007	0.01	0.017
N_5Cl_1-N_4(NNN)	0.232	0.009	0.007	0.016
N_5Cl_1-N_3Cl_1(ClNN)	0.288	0.007	0.007	0.015
N_2Cl_2-N_2Cl_2(ClN)	0.483	0.003	0.012	0.016
N_2Cl_2-N_3Cl_1(ClN)	0.486	0.005	0.011	0.016
N_2Cl_2-N_1Cl_3(ClN)	0.411	0.003	0.004	0.006
N_2Cl_2-N_1Cl_3(ClCl)	0.461	0.013	0.021	0.034
N_2Cl_2-N_2Cl_2(NN)	0.32	0.004	0.001	0.005
N_3Cl_1-N_3Cl_1(NN)	0.32	0.002	0	0.002
N_3Cl_1-N_1Cl_3(ClN)	0.43	0.003	0.004	0.007
N_3Cl_1-N_2Cl_2(ClN)	0.488	0.003	0.007	0.01
N_1Cl_3-N_2Cl_2(ClN)	0.41	0.003	0.004	0.007
N_1Cl_3-N_1Cl_3(ClCl)	0.525	0.012	0.033	0.045
N_2Cl_2-N_4(NN)	0.43	0.007	0.001	0.008
N_1Cl_3-Cl_4(ClCl)	0.5	0.006	0.03	0.036
N_1Cl_3-N_3Cl_1(ClN)	0.433	0.003	0.003	0.006
N_1Cl_3-N_2Cl_2(ClCl)	0.531	0	0.531	0.04
N_2Cl_2-N_3Cl_1(NN)	0.375	0.004	0.001	0.005
N_2Cl_2-Cl_4(ClCl)	0.546	0.006	0.016	0.022
N_3Cl_1-N_3Cl_1(ClN)	0.457	0.009	0.014	0.022
Cl_4-N_1Cl_3(ClCl)	0.406	0.005	0.016	0.021
Cl_4-Cl_4(ClCl)	0.322	0.004	0.003	0.007
Cl_4-N_2Cl_2(ClCl)	0.412	0.008	0.019	0.027
N_3Cl_1-N_2Cl_2(NN)	0.366	0.005	0.002	0.006
N_4-N_2Cl_2(NN)	0.419	0.005	0.005	0.01
N_4-N_3Cl_1(NN)	0.328	0.003	0.001	0.004
N_4-N_4(NN)	0.321	0.003	0.002	0.005
N_1Cl_3-N_1Cl_3(ClN)	0.294	0.002	0	0.003
N_3Cl_1-N_4(NN)	0.333	0.003	0.002	0.005
N_2Cl_2-N_4Cl_2(ClNN)	0.384	0.004	0.001	0.005
N_2Cl_2-N_3Cl_3(ClClN)	0.464	0.004	0.003	0.007
N_2Cl_2-N_3Cl_3(ClNN)	0.359	0.003	0.004	0.007
N_2Cl_2-N_1Cl_5(ClClN)	0.379	0.006	0.009	0.014
N_3Cl_1-N_6(NNN)	0.329	0.005	0	0.005
N_3Cl_1-N_3Cl_3(ClNN)	0.322	0.003	0.001	0.004
N_1Cl_3-N_2Cl_4(ClClN)	0.344	0.003	0	0.004
N_1Cl_3-N_1Cl_5(ClClCl)	0.49	0.007	0.018	0.025
N_1Cl_3-N_4Cl_2(ClClN)	0.433	0.009	0.005	0.014
N_2Cl_2-N_2Cl_4(ClClN)	0.398	0.005	0.007	0.012
N_2Cl_2-N_5Cl_1(ClNN)	0.408	0.007	0.001	0.008
N_2Cl_2-N_2Cl_4(ClNN)	0.333	0.007	0.001	0.008
N_1Cl_3-N_3Cl_3(ClClN)	0.362	0.003	0.002	0.006

Cl_4-N_2Cl_4(ClClCl)	0.341	0.005	0.006	0.011
Cl_4-N_1Cl_5(ClClCl)	0.277	0.003	0.002	0.005
N_3Cl_1-N_4Cl_2(ClNN)	0.338	0.006	0	0.006
N_4-N_3Cl_3(NNN)	0.329	0.006	0.001	0.007
N_4-N_5Cl_1(NNN)	0.288	0.006	0.003	0.009
N_1Cl_3-N_1Cl_5(ClClN)	0.307	0.004	0.001	0.005
N_3Cl_1-N_4Cl_2(NNN)	0.352	0.006	0.001	0.007
N_3Cl_1-N_2Cl_4(ClNN)	0.331	0.004	0.001	0.004
N_4-N_4Cl_2(NNN)	0.293	0.005	0.003	0.008
Cl_4-N_3Cl_3(ClClCl)	0.351	0.01	0.018	0.027
N_3Cl_1-N_5Cl_1(NNN)	0.352	0.008	0.005	0.014
N_2Cl_2-N_4Cl_2(ClClN)	0.468	0.007	0.003	0.01
N_1Cl_3-N_2Cl_4(ClClCl)	0.472	0.012	0.012	0.024
N_1Cl_3-N_3Cl_3(ClClCl)	0.588	0.001	0.588	0.04
N_3Cl_1-N_5Cl_1(ClNN)	0.348	0.009	0.005	0.014
Cl_6-Cl_4(ClClCl)	0.279	0.005	0.001	0.006
Cl_6-N_1Cl_3(ClClCl)	0.403	0.007	0.004	0.011
N_6-N_4(NNN)	0.234	0.007	0.012	0.019
N_2Cl_2-N_2Cl_2(ClCl)	0.58	0.009	0.013	0.022
Cl_4-Cl_6(ClClCl)	0.236	0.004	0.001	0.005
N_1Cl_3-Cl_6(ClClCl)	0.479	0.008	0.014	0.022
N_4-N_6(NNN)	0.285	0.006	0.005	0.011
N_3Cl_3-N_3Cl_1(NNN)	0.248	0.005	0.005	0.01
N_3Cl_1-N_3Cl_3(NNN)	0.344	0.009	0.007	0.016

Supplementary Note 1: Potential small occupation of octahedral sites

For the structure solutions shown in Tables S1-4 and Figure S2 all Li was assumed occupy tetrahedral sites as our energetic considerations show that tetrahedral sites are clearly more stable and based on the structure solution that was previously obtained for Li_5NCl_2 i.e. $Li_{1.66}Cl_{0.66}N_{0.33}$. [2]

However, our energetic considerations would permit a small occupation of the octahedral sites at 300K. The energy difference between octahedral and tetrahedral sites may be estimated from tet-oct and oct-tet jump-Ea values. For the $Li_{1+2x}Cl_{1-x}N_x$ phases the oct sites are destabilized by on average 0.07 ± 0.01 eV versus the tetrahedral sites. According to the partition function this would result in 3±1 % of Li ions occupying octahedral sites at 300 K:

$$q = 8 + 4 * \exp\left(\frac{-\Delta\epsilon_{site}(oct,tet)}{kT}\right) \quad \text{(S1)}$$

Where q is the partition function, ΔE_{site} (oct,tet) is the average energy difference between tet and oct sites, k is Boltzmann constant T is temperature.

$$p_{oct} = \frac{4 * \exp\left(\frac{-\Delta\epsilon_{site}\,(oct,tet)}{kT}\right)}{q} \quad (S2)$$

where p_{oct} is the of Li occupying the octahedral sites.

We investigated how the quality of Rietveld fits would vary with Li occupying octahedral sites. As a representative of the $Li_{1+2x}Cl_{1-x}N_x$ phase we use the $Li_{1.66}Cl_{0.66}N_{0.33}$ phase. In Figure S14 we report on Rietveld fits of the x-ray diffraction pattern of a $Li_{1.66}Cl_{0.66}N_{0.33}$ phase assuming 0%, 3%, 5%, 10%, 20%, 40% and 60% of Li occupying the octahedral sites. Between 0% and 5% the quality of fit (as determined by the R_{wp} and χ^2 fit parameters) is hardly altered. From 10% of Li occupying octahedral sites onwards the fit quality gradually worsens. We performed the same investigation on a Neutron diffraction pattern of $Li_{1.66}Cl_{0.66}N_{0.33}$ which was additionally annealed to 600 °C for 3h and quenched in ambient temperature to improve crystallinity Figure (S15). The conclusions drawn from Figure S15 are equivalent: Between 0% and 5% the quality of fit (as determined by the R_{wp} and χ^2 fit parameters) is hardly altered. From 10% of Li occupying octahedral sites the fit quality gradually worsens. This investigation experimentally supports that the tetrahedra are indeed more stable than the octahedral sites (as our energetic considerations from DFT clearly demonstrate) however ~3% of Li ions occupying the octahedral sites may not be confirmed or excluded from neutron and x-ray Rietveld refinements and is thus conceivable. We note that a potentially (~3%) of Li ions occupying octahedral sites would not alter any of the conclusions of this study.

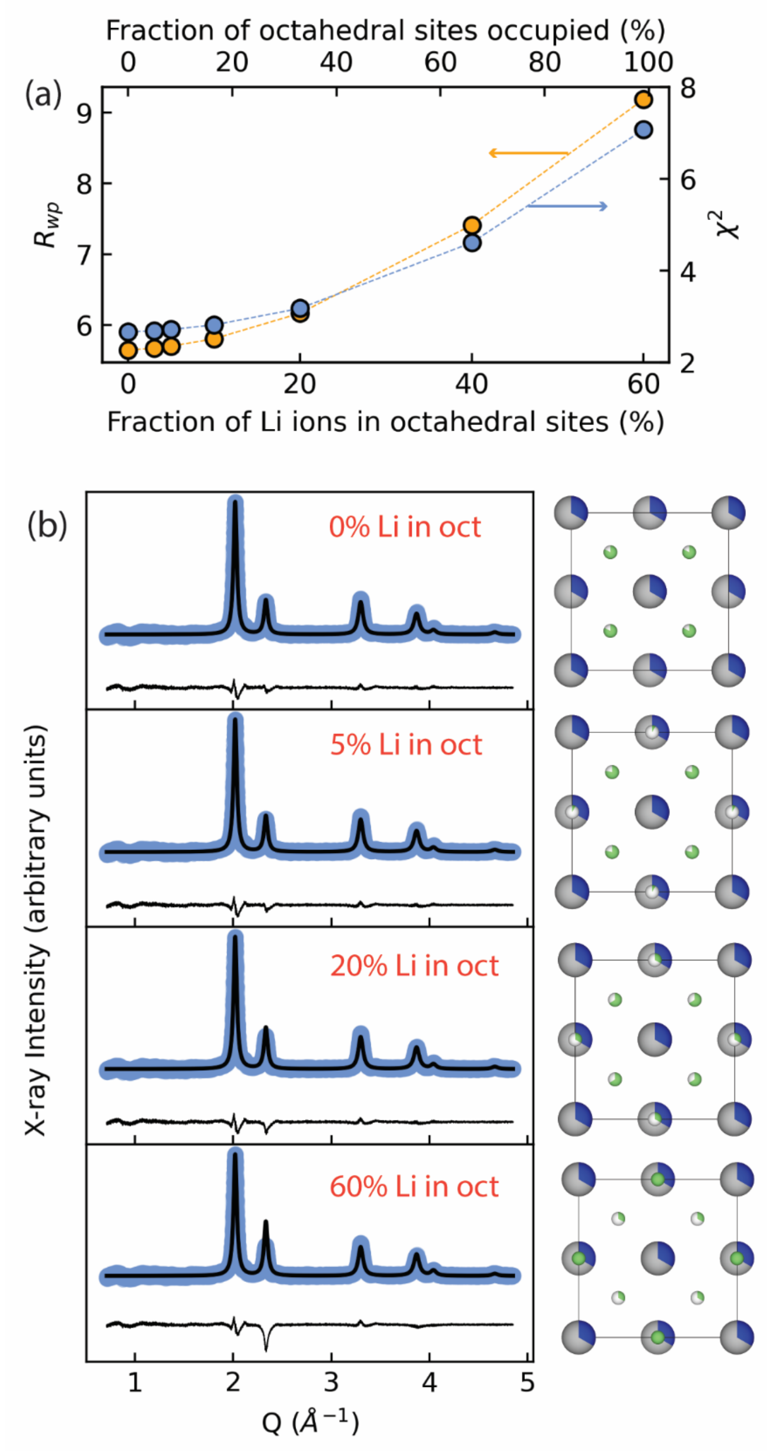

Figure S15. Rietveld refinements of a $Li_{1.66}Cl_{0.66}N_{0.33}$ x-ray diffraction patterns with different fractions of Li-ions occupying the tet and oct sites. (a) R_{wp} and χ^2 fit parameters as a function of the fraction of Li ions occupying the oct and tet sites respectively. (b) Rietveld fits with increasing fraction of Li ions occupying the oct sites along with the respective unit cells viewed along the [001] axis.

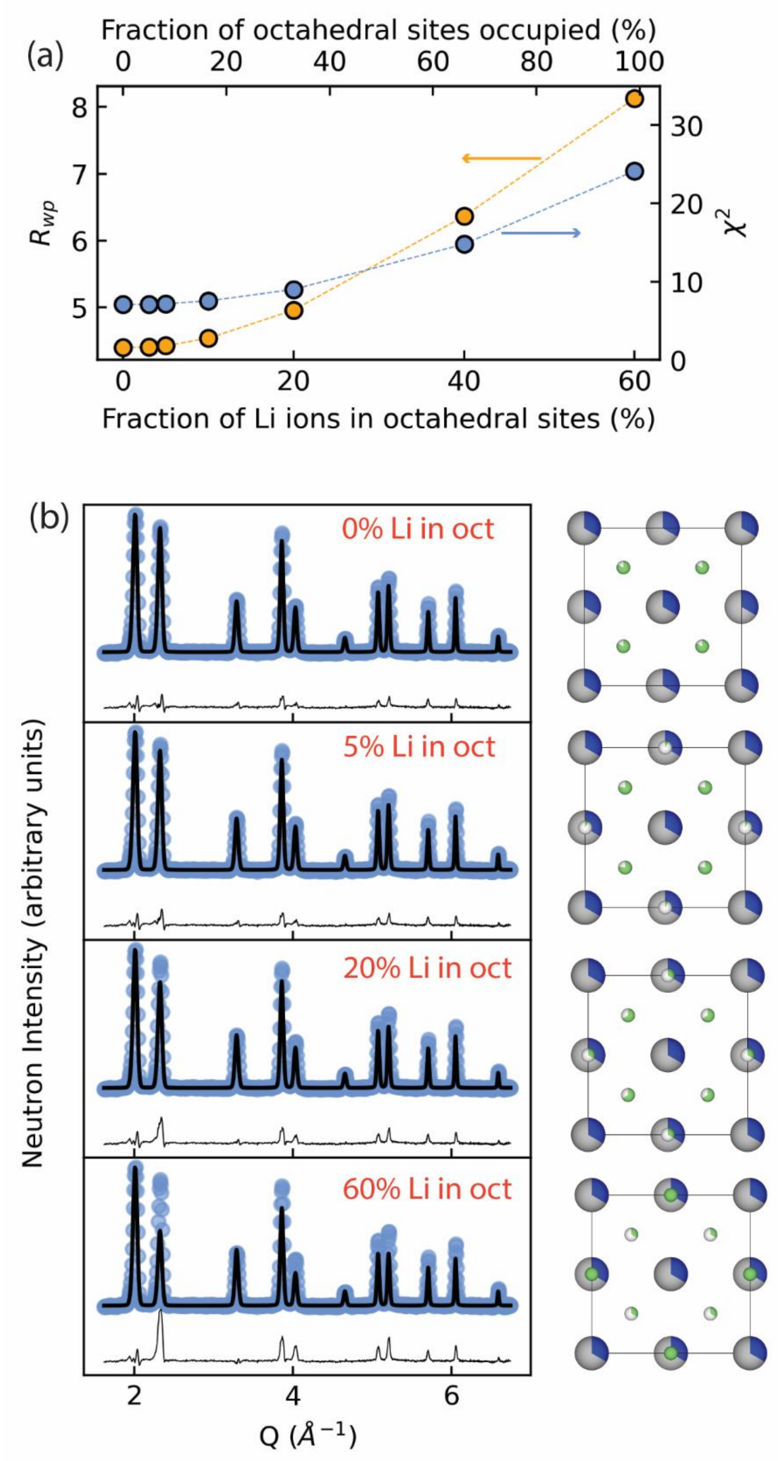

Figure S16. Rietveld refinements of a $Li_{1.66}Cl_{0.66}N_{0.33}$ neutron diffraction patterns with different fractions of Li-ions occupying the tet and oct sites. (a) R_{wp} and χ^2 fit parameters as a function of the fraction of Li ions occupying the oct and tet sites respectively. (b) Rietveld fits with increasing fraction of Li ions occupying the oct sites along with the respective unit cells viewed along the [001] axis.

Supplementary Note 2: tetrahedral sites in LiCl – sites or 'merely' positions ?

Catlow et al. proposed conditions that must be fulfilled so that ion diffusion in solids may be interpreted as ion-hops between well-defined sites.[3] For crystallographic positions to qualify as sites Catlow et al. propose that the following conditions laid out in Table S8 need to be satisfied. Whether these conditions are fulfilled for tetrahedral and octahedral positions in LiCl was investigated via the AIMD simulations of LiCl. The results are reported in Table S9. As clearly identified in Table S9 the octahedral positions in LiCl from AIMD simulations fulfil all conditions to qualify as sites while the tetrahedral positions do not. The tetrahedral site should thus arguably more generally be referred to as 'positions' than 'sites'. In this study however, we refer to both the octahedral and tetrahedral positions as sites while highlighting the high metastability of the tetrahedral position in LiCl in the main text.

Table S8 shows the requirements for crystallographic positions to qualify as sites defined by Catlow et al. [3] Tr stands for time spent in site, Th stands for time of hopping between sites, v* stands for the attempt frequency d stands for the distance between two sites and a for the vibrational amplitude in the site. ΔE_{hop} stands for the hop activation energy. All these properties may be extracted from AIMD simulations.

**Property**
$T_r >> T_h$
$Tr >> (v^*)^{-1}$
$d >> a$
$\Delta E_{hop} >> kT$

Table S9 shows whether the requirements defined in Table S8 are fulfilled for oct and tet sites in LiCl obtained from AIMD simulation of LiCl.

**Property**	**Oct-site**	**Tet site**	**Conditions for site fulfilled [oct,tet]**
T_r/T_h	892.8	0.12	[Yes, No]
$Tr/(v^*)^{-1}$	1863	0.26	[Yes, No]
d/a	5.79	5.79	[Yes, Yes]
$\Delta E_{hop}/kT$	25	1.2	[Yes, No]

Supplementary Note 3: Calculating the max ion radius that fits in a tetrahedral site

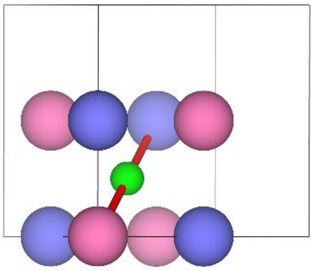

Figure S17 . Schematic of how the maximum ion radius fitting in a specific tetrahedral site may be obtained. An ion fitting in the tetrahedron spanned by the blue spheres also fits in a tetrahedron spanned by the pink spheres so that the cube consisting of blue/grey and pink spheres may be used to calculate the max ion radius fitting in a tetrahedral site. Note this method only works for tetrahedra where the ions at the corners are all the same. The tetrahedral site is indicated by a green sphere. The red line is the cube body diagonal.

An ion fitting in a tetrahedral site spanned by the blue spheres in Figure S16 also fits in the tetrahedron spanned by the pink spheres (i.e. rotated by 90 degrees) and thus the cube spanned by the pink and blue spheres may be used to calculate the max ion radius that fits in a site. This method only for tetrahedra where the ions at the corner of the tetrahedron are all the same. The maximum ion radius (r_{void}) fitting in a tetrahedron may be calculated as such

$$d = 2 * (\, r_{void} + r_{peripheral}) \quad \text{(S3)}$$

$$r_{void} = \frac{d}{2} - \, r_{peripheral} \quad \text{(S4)}$$

Where $r_{peripheral}$ is the radius of the ions at the corner of the tetrahedron (Cl^- or N^{3-}) and d the body diagonal of the cube spanned by the positions of the peripheral ions and the periphareal ion-positions rotated by 90 around an arbitrary rotation axis going through the tetrahedron centre (Figure S16) (i.e. blue and pink spheres)

For LiCl this results in r_{void} = 0.38 Å by estimating d from the unit cell of LiCl. Table S shows different r_{void} radii calculated for different phases.

Table S10. Max radii fitting in tetrahedra of LiCl and $Li_{1+2x}Cl_{1-x}N_x$ phases

Phase	Lattice param (Å)	Tertrahedron type	r_{void} (Å)
LiCl	5.15	Cl_4	0.38
$Li_{1+2x}Cl_{1-x}N_x$ (0.33<x<0.5)	5.36 (on average)	Cl_4	0.48
$Li_{1+2x}Cl_{1-x}N_x$ (0.33<x<0.5)	5.36 (on average)	N_4	0.84
$Li_{1+2x}Cl_{1-x}N_x$ (0.33<x<0.5)	5.36 (on average)	Cl_3N_1, Cl_2N_2, Cl_1N_3	$0.48 < r_{void} < 0.84$

Supplementary Note 4: More details on the metastability calculations

Phase diagrams for the metastability calculations were built by leveraging the materials project database. The phases in table S11 are thermodynamically stable in the materials project database [4] on the LiCl-Li_3N and Li_3N Li_2S tielines. In the materials project database the Li_9S_3N (mp557964) phase is slightly metastable (0.008 eV/atom above the hull). We lowered the energy of this phase to the hull since previous experimental work showed that this phase is thermodynamically stable and should be considered in phase diagrams. [1,5]

Table S11. Entries taken from the materials project database

Thermodynamically stable phases (space group)	Material's project ID of computed entry
LiCl (Fm-3m)	mp1185319
Li_2S (Fm-3m)	mp1153
Li_3N (P6/mmm)	mp2251
Li_4NCl (R -3 m h)	mp29149
Li_9S_3N (Pm-3m)	mp557964

The reactions for the LiCl-Li_3N tieline where, in the equations below $Li_{1+2x}Cl_{1-x}N_x$ refers to antifluorite-like phases and Li_4NCl refers to the R-3mh phase:

$$Li_{1+2x}Cl_{1-x}N_x \rightarrow (1-2x)LiCl + x\,Li_4NCl \quad (for\ 0 < x < 0.5) \quad \text{(S5)}$$

$$Li_{1+2x}Cl_{1-x}N_x \rightarrow (1-x)\,Li_4NCl + (2x-1)Li_3N \quad (for\ x > 0.5) \quad \text{(S6)}$$

The reactions for the Li_2S-Li_3N tieline where in the equations below $Li_{2+x}S_{1-x}N_x$ refers to antifluorite-like phases and Li_9S_3N refers to the Pm-3m phase:

$$Li_{2+x}S_{1-x}N_x \rightarrow (4x-1)\,Li_2S + x\,Li_9S_3N \quad (for\ 0 < x < 0.25) \quad \text{(S7)}$$

$$Li_{2+x}S_{1-x}N_x \rightarrow \frac{1}{3}(1-x)\,Li_9S_3N + \frac{1}{3}(4x-1)Li_3N \quad (for > 0.25) \quad \text{S(8)}$$

References

1. Landgraf, V. *et al.* Entropy-induced high conductivity in fully-reduced electrolytes for solid-state batteries with lithium metal anodes. *ChemRxiv* (2023).

2. Marx, R. & Mayer, H. M. Preparation and Crystal Structure of Ordered and Disordered Lithium Nitride Dichloride, Li5NCl2. *J. Solid State Chem.* **130**, 90–96 (1997).

3. Catlow, C. R. A. Static lattice simulation of structure and transport in superionic conductors. *Solid State Ionics* **8**, 89–107 (1983).

4. Jain, A. *et al.* Commentary: The materials project: A materials genome approach to accelerating materials innovation. *APL Mater.* **1**, 001002 (2013).

5. Marx, R., Lissner, F. & Schleid, T. $Li_9N_2S_3$: Das erste Nitridsulfid der Alkalimetalle in einer Li2O-Typ-Variante. *Zeitschrift für Anorg. und Allg. Chemie* **632**, 2151 (2006).
